# Structural, Spectroscopic, and Computational Insights from Canavanine-Bound and Two Catalytically Compromised Variants of the Ethylene-Forming Enzyme

**DOI:** 10.1021/acs.biochem.4c00031

**Published:** 2024-04-05

**Authors:** Shramana Chatterjee, Matthias Fellner, JoelA. Rankin, Midhun G. Thomas, Simahudeen Bathir J S Rifayee, Christo Z. Christov, Jian Hu, Robert P. Hausinger

**Affiliations:** † Department of Microbiology, Genetics, and Immunology, Michigan State University, East Lansing, Michigan 48824, United States; ‡ Department of Biochemistry and Molecular Biology, Michigan State University, East Lansing, Michigan 48824, United States; § Department of Chemistry, Michigan Technological University, Houghton, Michigan 49931, United States; ∥ Department of Chemistry, Michigan State University, East Lansing, Michigan 48824, United States

## Abstract

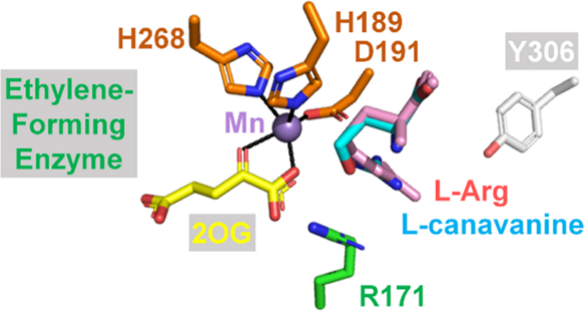

The ethylene-forming enzyme (EFE) is an Fe(II), 2-oxoglutarate (2OG), and l-arginine (l-Arg)-dependent oxygenase that either forms ethylene and three CO_2_/bicarbonate from 2OG or couples the decarboxylation of 2OG to C5 hydroxylation of l-Arg. l-Arg binds with C5 toward the metal center, causing 2OG to change from monodentate to chelate metal interaction and OD1 to OD2 switch of D191 metal coordination. We applied anaerobic UV–visible spectroscopy, X-ray crystallography, and computational approaches to three EFE systems with high-resolution structures. The ineffective l-Arg analogue l-canavanine binds to the EFE with O5 pointing away from the metal center while promoting chelate formation by 2OG but fails to switch the D191 metal coordination from OD1 to OD2. Substituting alanine for R171 that interacts with 2OG and l-Arg inactivates the protein, prevents metal chelation by 2OG, and weakens l-Arg binding. The R171A EFE had electron density at the 2OG binding site that was identified by mass spectrometry as benzoic acid. The substitution by alanine of Y306 in the EFE, a residue 12 Å away from the catalytic metal center, generates an interior cavity that leads to multiple local and distal structural changes that reduce l-Arg binding and significantly reduce the enzyme activity. Flexibility analyses revealed correlated and anticorrelated motions in each system, with important distinctions from the wild-type enzyme. In combination, the results are congruent with the currently proposed enzyme mechanism, reinforce the importance of metal coordination by OD2 of D191, and highlight the importance of the second coordination sphere and longer range interactions in promoting EFE activity.

## Introduction

**Scheme 1:**
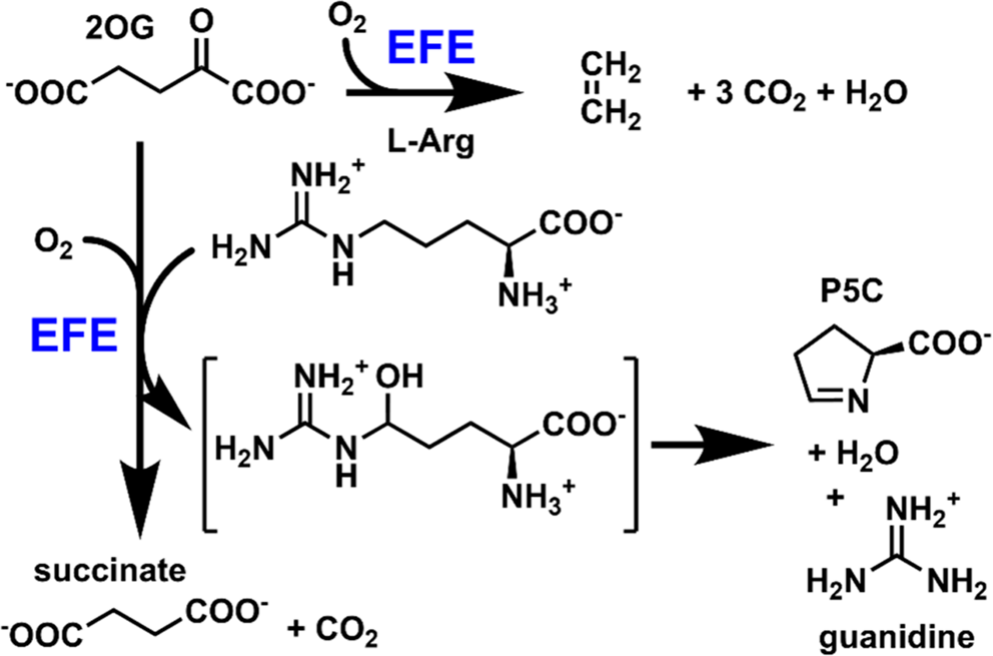
Major Reactions of the EFE

Some bacteria and fungi possess an Fe(II), 2-oxoglutarate (2OG), and l-arginine (l-Arg)-dependent oxygenase known as the ethylene-forming enzyme (EFE)^1^ that (1) cleaves three C–C bonds of 2OG to form ethylene and three molecules of CO_2_/bicarbonate and (2) catalyzes the oxidative decarboxylation of 2OG to form CO_2_ and succinate coupled to the C5 hydroxylation of l-Arg, which decomposes to guanidine and l-Δ^1^-pyrroline-5-carboxylate (P5C) (Scheme 1).^2,3^ Computational and biochemical studies suggest that the bifurcated pathway splits at the level of an Fe(III)·superoxo complex, with the major reaction, ethylene generation, requiring dioxygen insertion into the C1–C2 bond of 2OG, formation of a propionyl-3-yl radical and an Fe(III)-bound carbonate, and their coupling followed by fragmentation to yield the target product.^4−7^ In contrast, the minor reaction of guanidine/P5C formation is associated with the well-known hydroxylation chemistry of 2OG-dependent oxygenases that involves a ferryl intermediate.^1,8^

The best characterized EFE is that from the *Pseudomonas savastanoi* (formerly *Pseudomonas syringae*) pv *phaseolicola* strain PK2, for which several crystal structures have been reported.^8−10^ This enzyme binds mononuclear Fe(II) using three side chains (H189, D191, and H268) with three water molecules completing the six-coordinate geometry. Unlike the situation reported for several other 2OG-dependent oxygenases where 2OG displaces two coordinating waters and chelates the Fe(II) using both its C1 carboxylate and C2 keto group, 2OG binds to Fe(II) of the EFE predominantly in a monodentate mode with just its C1 carboxylate.^3,9^l-Arg addition induces 2OG to adopt the conventional chelate binding mode, while also causing dissociation of the remaining coordinated water molecules and formation of a twisted peptide bond between the metal ligand D191 and the neighboring residue Y192.^9^ The resulting open coordination site for oxygen binding is directed away from l-Arg (i.e., an “off-line” binding mode).

Here, we extend our understanding of EFE catalysis by carrying out biochemical, structural, and computational studies of the enzyme in complex with a substrate analogue and using two enzyme variants. First, we consider the binding of l-canavanine, a chemical analogue of l-Arg in which the C5 methylene group is replaced by an oxygen atom. The EFE cannot transform this analogue into guanidine and P5C, but we also showed that the l-canavanine-bound enzyme fails to induce ethylene generation.^3^ To understand this lack of reactivity, we used anaerobic ultraviolet–visible (UV–vis) spectroscopy to examine the effect of l-canavanine on the metal-to-ligand charge-transfer (MLCT) transitions of EFE·Fe(II)·2OG and determined the structure of the EFE·Mn(II)·2OG·l-canavanine complex. Second, we examined the R171A variant of the EFE,^9^ a substitution of a second-sphere residue (i.e., just beyond the immediate metal ligands) that eliminates ethylene-forming activity likely by destabilizing 2OG binding. We carried out MLCT analysis and elucidated the R171A EFE crystal structure to provide evidence for significant changes in the 2OG binding pocket that explain the abolishment of activity, and we used electrospray ionization–mass spectrometry (ESI-MS) to identify an unanticipated ligand at the 2OG binding site. Third, we focused on the nearly inactive Y306A variant of the EFE for which the substitution occurs distant from the active site.^9^ We noted distinct behavior when investigating the MLCT transitions associated with Y306A EFE·Fe(II)·2OG and obtained insights from the structure of the Y306A EFE·Mn(II)·2OG complex that was crystallized in the presence of unbound l-Arg. Crystallographic structures can provide only partial insights into flexibility^11^ even though enzymes exhibit a broad range of complex motions that can be functionally important.^12−14^ Many examples illustrate the role of the second coordination sphere in such long-range interactions,^15−20^ and the flexibility and stability of different parts of the enzyme can be crucial to understand changes in the catalytic reactivity.^14,21−25^ Thus, we complemented the biochemical and structural results by applying molecular dynamics (MD) simulations^26−29^ to explore the conformational changes and substrate or substrate analogue interactions for all three EFE systems.

## Materials and Methods

### Chemicals

l-Canavanine, tris(2-carboxyethyl)phosphine (TCEP), l-Arg, sodium phosphate monobasic, and imidazole were acquired from Sigma (St. Louis, Missouri). Kanamycin, isopropyl β-d-1-thiogalactopyranoside (IPTG), 4-(2-hydroxyethyl)-1-piperazineethanesulfonic acid (HEPES), and Ni-NTA agarose beads were purchased from GoldBio (St. Louis, Missouri). 2OG was purchased from Fluka. EDTA was purchased from Invitrogen. 50% PEG 3350, PEG 400, PEG MME 550, and 50% ethylene glycol were purchased from Rigaku or Hampton. All other chemicals were reagent grade or better.

### Enzyme Purification

Wild-type (WT) *P. savastanoi* strain PK2 EFE (Uniprot entry P32021) and its R171A and Y306A variants were obtained as previously described.^9^ Briefly, the genes encoding N-terminal His_6_-tagged proteins were expressed in recombinant *Escherichia coli* cells that were grown in Luria broth or Terrific broth and induced with IPTG; the cells were disrupted by use of a French pressure cell or sonication, the proteins were purified by use of nickel-nitrilotriacetic acid (NTA) resin, the tag was cleaved by using His_7_-tagged tobacco etch virus (TEV) protease, and the proteins were rechromatographed on the nickel-NTA column to provide the final samples.

### Enzyme Assays

The production of ethylene was monitored by gas chromatography, and the formation of P5C was assessed by reaction with 2-aminobenzaldehyde as previously described.^3^

### UV–vis Spectroscopy

Anaerobic UV–vis spectroscopy was used to probe the MLCT transitions associated with 2OG coordinated to the EFE Fe(II) center via the chelate mode.^3,30^ All solutions were prepared anaerobically in 25 mM HEPES buffer that was adjusted to pH 8.0. Spectra were recorded at room temperature using a Shimadzu UV-2600 spectrophotometer.

### Crystallization

The WT EFE (0.5 μL of 64 mg of protein mL^–1^) was incubated for 1 h on ice with 25 mM HEPES (pH 8.0) buffer containing 1 mM MnCl_2_, 1 mM TCEP, 3 mM l-canavanine, and 2 mM 2OG before setting up crystallization drops with 0.5 μL of reservoir solution at 4 °C. The sitting drop reservoir of 200 μL contained 25% w/v poly(ethylene glycol) 3350, 0.1 M Bis-Tris (pH 6.5), and 0.2 M sodium chloride. The resulting crystals were soaked for about 1 min in 25% w/v poly(ethylene glycol) 400 and 75% reservoir solution before freezing.

The R171A variant of the EFE in 25 mM HEPES (pH 8.0) buffer containing 1 mM TCEP was crystallized in a similar manner with a sitting drop reservoir containing 20% w/v poly(ethylene glycol) 3350 and 0.2 M sodium citrate tribasic dihydrate. The crystals were briefly soaked in 25% poly(ethylene glycol) 550 monomethyl ether and 75% reservoir solution before freezing.

The Y306A variant of the EFE (0.5 μL of 64 mg mL^–1^) was incubated in 25 mM HEPES (pH 8.0) buffer containing 1 mM MnCl_2_, 1 mM TCEP, 3 mM l-Arg, and 3 mM 2OG before mixing with 0.5 μL of reservoir solution. The sitting drop reservoir of 200 μL contained 25% w/v poly(ethylene glycol) 3350, 0.1 M Bis-Tris (pH 6.5), and 0.2 M sodium chloride. The resulting crystals were soaked for 5 min in 25% w/v ethylene glycol and 75% reservoir solution before freezing.

### Structure Determinations

X-ray diffraction data were collected at the Advanced Photon Source LS-CAT beamline 21-ID-D. For details, see Table S1. Datasets were indexed and integrated with HKL-2000 or iMosflm,^31^ and merging and scaling were done using Aimless.^32^ Molecular replacement and refinement were done in Phenix^33^ with model building in COOT.^34^ Datasets were uploaded to the Protein Data Bank (PDB) with IDs of 6CBA for EFE·Mn(II)·2OG·l-canavanine, 6CF3 for the Y306A variant of EFE·Mn(II)·2OG, and 8UC2 for the R171A variant of EFE·Ni(II)·benzoate.

### ESI-MS

R171A EFE crystals were recovered from the drop, cleaned, and dissolved in 10 mM HEPES, pH 8.0. A fourfold volume of acetonitrile was added to precipitate the protein, which was removed by centrifugation, and the samples were dried and dissolved in water containing 0.1% formic acid. Standards (benzoic acid, nicotinic acid, and salicylic acid) were similarly prepared from ACS reagents in water containing 0.1% formic acid. The samples and standards were injected onto a cyano-chemistry HPLC column that was equilibrated in 0.1% formic acid and eluted with an increasing gradient of acetonitrile. The fractions were analyzed by ESI-MS using a XEVO G2-XS instrument in negative ionization mode.

### System Preparation for MD Simulations

As starting structures for the EFE·Fe(III)·OO^•–^ complexes of R171A and Y306A variants, the crystal structure of the WT EFE complex with Mn(II), 2OG, and l-Arg (PDB: 5V2Y) was used.^9^ Mn(II) was substituted with Fe(II), and dioxygen was coordinated to the Fe(II) center. The crystal structure of the EFE variant Y306A (PDB: 6CF3) with Mn(II) and 2OG was used as a starting structure for the Y306A EFE·Fe(II)·2OG complex. The structure was modified by replacing the Mn(II) with Fe(II) using GaussView 5.0.^35^ The R171A and Y306A substitutions were generated using the Leap module of AMBER 18.^36^ The starting structures for the two EFE variants were generated similarly to that of the WT EFE.^5^ Hydrogen atoms were added according to the protonation states using Amber routines.^36^ The Antechamber module containing the GAFF tool was used to develop topology files of the nonstandard components such as 2OG and O_2_.^37,38^ Parameters for Fe(II) (high spin *S* = 2 and *M* = 5), coordinating 2OG, H189, D191, H268, and WAT503 and parameters for the Fe(III) center (high spin *S* = 2, *M* = 5 after ferromagnetic coupling between the iron and dioxygen) coordinating 2OG, H189, D191, H268, and dioxygen were generated using Metal Center Parameter Builder (MCPB v3.0).^39^ The rest of the protein was modeled using the FF14SB force field.^40^ The system was solvated with a 10 Ȧ water box from the farthest point of the residue, and counterions (Na^+^) were introduced to neutralize the system.

For the simulation of the EFE containing l-canavanine, we used the crystal structure of the WT EFE containing Mn(II), 2OG, and l-Arg (PDB: 5V2Y)^9^ and replaced the l-Arg coordinates with those for l-canavanine obtained from the EFE·Mn(II)·2OG·l-canavanine crystal structure (PDB: 6CBA). The two crystal structures are highly superimposable; therefore, the WT structure with l-canavanine was used for the simulations. The remaining steps of system preparation for the EFE·Fe(III)·OO^•–^·l-canavanine complex followed the same procedure as used for the two variants.

### MD Simulations

The protonation states were assigned considering the p*K*_a_ of the residues at neutral pH with the tLeAP module of AMBER.^36^ In particular, E84 is deprotonated, Arg171 is protonated, and the guanidino groups of l-canavanine and l-Arg are protonated, while their carboxyl groups are deprotonated. The procedure for MD simulations was the same as that used for the WT EFE.^5^ Initially, the modeled systems were subjected to minimizations in two steps. The first minimization was performed with a restraint weight of 100 kcal mol^–1^ on the solute molecule with 5000 steps of the steepest descent and 5000 steps of the conjugate gradient. The second minimization was performed similarly on the full system without any restraint. The minimizations were performed using the CPU version of AMBER 18 (SANDER).^36^ Minimizations were followed by the gradual heating of the system at the NVT ensemble from 0 to 300 K using a Langevin thermostat for 250 ps.^41^ The collision frequency of the Langevin thermostat was set to 1 ps^–1^. During the heating, the solute molecules were restrained using a harmonic potential of 50 kcal mol^–1^. After heating, 1 ns simulations with weak restraint on the protein were run to achieve uniform density. At a constant pressure of 1 bar, MD was equilibrated for all systems for 3 ns. Constant pressure was achieved by employing a Berendsen barostat. From the equilibrated structure, 1 μs of MD simulation was performed with a 2 fs time step. The particle mesh Ewald (PME) method^42^ was employed to compute long-range interactions within a 10 Å cutoff in all simulations. The simulations were conducted utilizing the GPU-accelerated version of AMBER–PMEMD. Hydrogen bonding interactions were analyzed with CPPTRAJ.^43^ Principal component analysis (PCA), dynamic cross-correlation analysis (DCCA), and differential DCCA (d-DCCA) (for analyzing the differences in correlated motions between two systems) were conducted on the Cα carbons of the protein residues using the last 500 ns trajectories, and these analyses were executed with the Bio3D module implemented in the R software package.^44^ Binding energies were calculated using molecular mechanics, the generalized Born model, and the solvent accessibility method (MMGBSA).^45,46^

## Results and Discussion

### Experimental Studies on the l-Canavanine-Bound EFE

**Figure 1:**
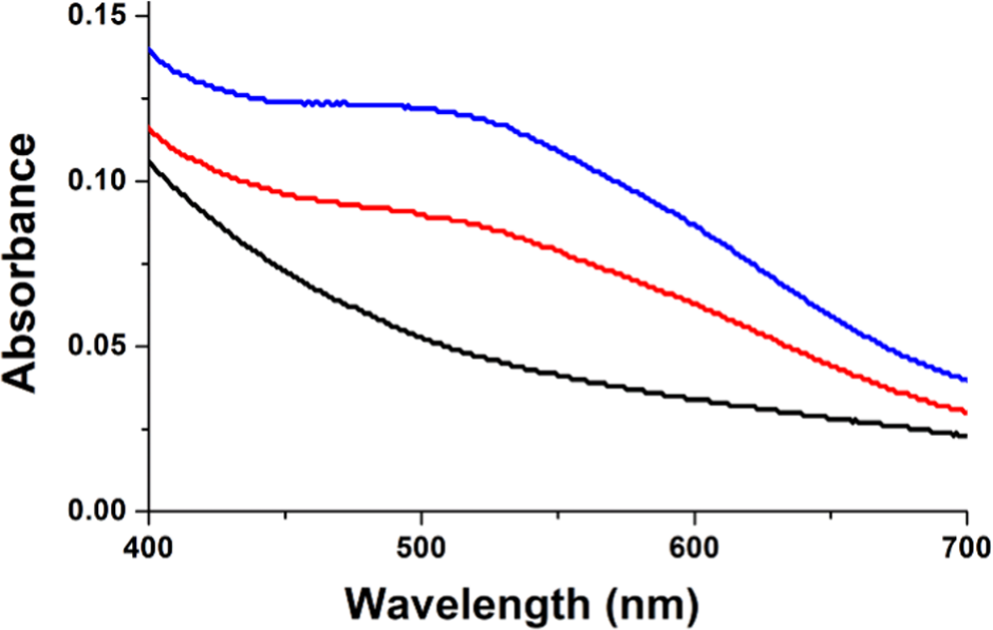
Anaerobic UV–vis spectra of EFE·Fe(II) (black), EFE·Fe(II)·2OG (red), and EFE·Fe(II)·2OG·l-canavanine (blue) complexes. The anaerobic samples contained 242 μM EFE, 1 mM Fe(II), and (when present) 2.5 mM 2OG and 2.5 mM l-canavanine. The spectra were adjusted for dilution.

As previously reported, the addition of 2OG to an anaerobic solution of EFE·Fe(II) generates a weak difference spectrum with a maximum absorption near 525 nm that was attributed to a mixture of monodentate and bidentate binding of 2OG to the metal, where only the chelated species provides the broad MLCT transitions.^3^ Subsequent addition of l-Arg shifts the equilibrium to favor the chelate binding mode that is essential for activity, as noted by the increased absorbance in this region.^3^ Very much like the situation for added l-Arg, the addition of l-canavanine to the EFE·Fe(II)·2OG sample leads to an intensification of the calculated difference absorption from ∼240 to ∼450 M^–1^ cm^–1^ (Figure 1). These results are consistent with the binding of l-canavanine leading to a shift from monodentate 2OG binding to Fe(II) to the chelate binding mode. In this case, the chelate binding of 2OG is insufficient for promoting the ethylene-forming activity of the enzyme.

To investigate why l-canavanine does not promote ethylene formation despite its ability to bind much like l-Arg according to the anaerobic spectroscopy evidence, we examined the structure of EFE·Mn(II)·2OG with bound·l-canavanine. We previously reported that six l-Arg analogues significantly reduced the ethylene-forming activity of the EFE when substituted for l-Arg.^3^ In particular, the relative amount of ethylene generated was reduced to ∼15% for *N*^γ^-hydroxy-l-Arg and 0.1–1% when using citrulline, homoarginine, agmatine, l-argininamide, and l-canavanine. We obtained no evidence for processing of these compounds (e.g., hydroxylation at C3 or C4 of canavanine was not detected). Our earlier studies revealed similar structures for EFE·Mn(II)·2OG·l-Arg (PDB: 5V2Y), EFE·Mn(II)·2OG·*N*^γ^-hydroxy-l-Arg (PDB: 5VKA), and EFE·Mn(II)·2OG·l-argininamide (PDB: 5VKB), despite the inability of the latter compounds to generate ethylene.^9^

**Figure 2:**
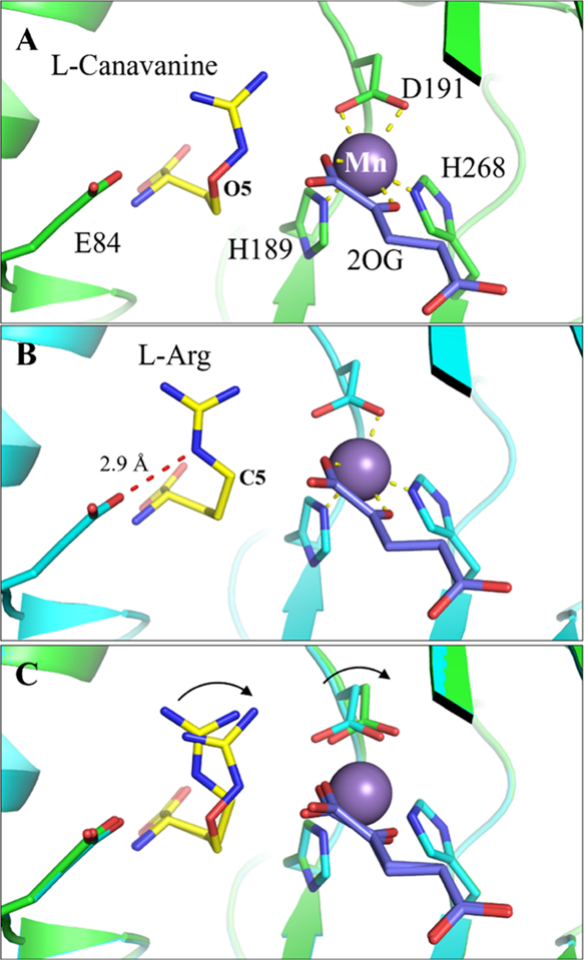
Structural comparison of the EFE·Mn·2OG·l-canavanine complex and the EFE·Mn·2OG·l-Arg complex. (A) Active site of the EFE with bound l-canavanine (PDB: 6CBA). (B) Active site of the EFE with bound l-Arg (PDB: 5V2Y). (C) Superimposition of the two structures. The curved arrows indicate the conformational changes from the l-Arg-bound structure to the l-canavanine-bound structure. The yellow dashed lines indicate metal coordination with Mn(II), whereas the red dashed lines show the hydrogen bonds between the bound ligand and E84.

**Figure 3:**
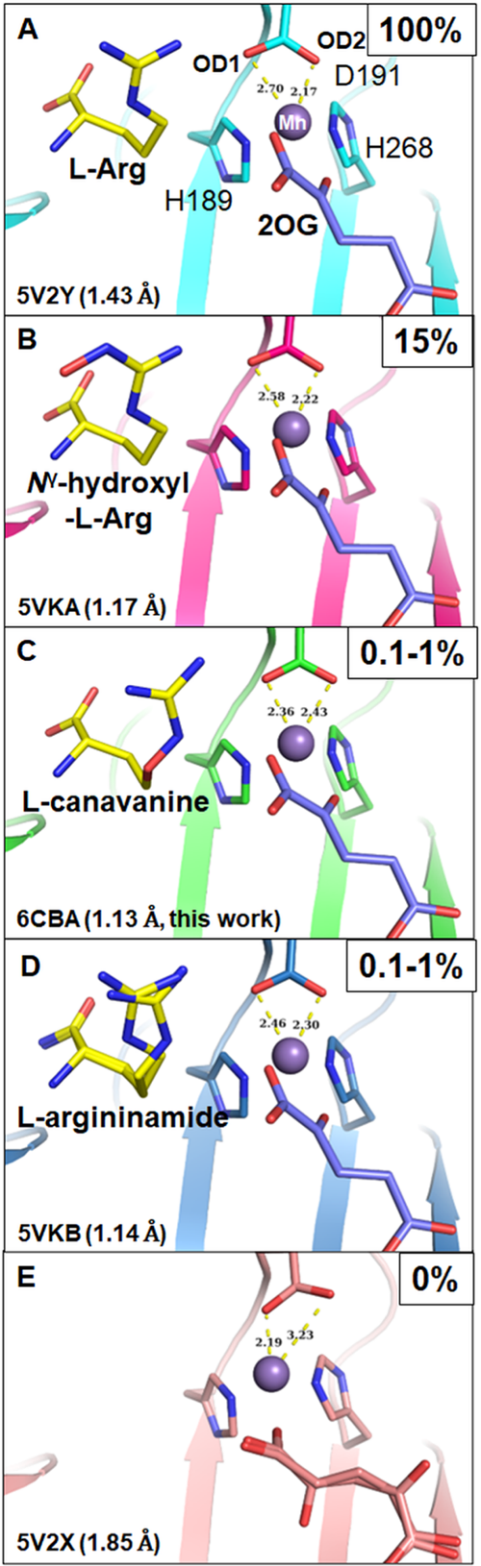
Metal coordination in the EFE without and with l-Arg or other l-Arg analogues. (A) EFE·Mn(II)·2OG·l-Arg complex. (B) EFE·Mn(II)·2OG·*N*^γ^-hydroxyl-l-Arg complex. (C) EFE·Mn(II)·2OG·l-canavanine complex. (D) EFE·Mn(II)·2OG·l-argininamide complex. (E) EFE·Mn(II)·2OG complex. The relative ethylene producing activity is shown in the upper right corner of each panel, and the PDB ID and resolution for each structure are shown in the lower left corner. The yellow dashed lines indicate the coordination distances (labeled in angstroms) between OD1/2 of D191 and Mn(II) (purple spheres). 2OG is illustrated in sticks with blue carbon atoms when chelating the metal and with pink carbon atoms when coordinating Mn in a monodentate manner.

The EFE·Mn(II)·2OG·l-canavanine structure was solved at 1.13 Å resolution (PDB: 6CBA) and shown to be highly superimposable (Cα RMSD of 0.07 Å) with the structure of the EFE·Mn(II)·2OG·l-Arg complex (PDB: 5V2Y), except that the bound l-canavanine adopts a distinct conformation from l-Arg (Figure 2). In the l-Arg-bound structure, C5 of l-Arg points toward Mn; in contrast, O5 of l-canavanine, which is equivalent to C5 of l-Arg, points away from the metal and toward E84. Close inspection of the density map excluded the possibility of the formation of an O5–E84 hydrogen bond because E84 is very likely in the deprotonated state. Nevertheless, l-canavanine adopts an orientation that leads to a substantial displacement of its guanidine group and accordingly a shift of the side chain of D191. This shift causes a change in the geometry of the metal coordination in the l-Arg-bound structure; the distances between Mn and OD1 and OD2 of D191 are 2.70 and 2.17 Å, respectively; in the l-canavanine-bound structure, however, the distances are 2.36 and 2.43 Å, respectively. As Mn exhibits monodentate coordination with OD1 in the absence of l-Arg (i.e., the EFE·Mn·2OG complex structure, PDB: 5V2X), the l-canavanine-bound structure represents an intermediate state between the l-Arg-unbound OD1 chelation and the l-Arg-bound OD2 chelation (Figure 3).

The l-canavanine-bound structure resembles the previously determined structures of the enzyme with l-Arg, *N*^γ^-hydroxy-l-Arg, and l-argininamide (Figure 3).^9^ As for the case of bound l-Arg, all these analogues induce a bidentate binding mode of 2OG, congruent with the anaerobic spectroscopy results. In addition, l-canavanine introduces the same side chain changes seen with the other analogues, as discussed in detail in our earlier work.^9^ For EFE·Mn(II)·2OG·*N*^γ^-hydroxy-l-Arg, the most active analogue (15% activity), the distances between Mn and OD1 and OD2 of D191 are 2.58 and 2.22 Å, respectively. This structure is nearly identical to that of the l-Arg-bound structure except for the extra *N*^γ^-hydroxyl group that prevents C5 hydroxylation, leading to a decrease of activity likely due to the subtle conformation change. The complex containing l-argininamide revealed two conformations of the substrate analogue, one matching l-Arg/*N*^γ^-hydroxy-l-Arg and the second shifting the C5 and the Nε atom with the guanidine group still forming a similar hydrogen bond to OD1 of D191. Only one conformation for D191 is observed in the structure with distances of 2.46 and 2.30 Å. When comparing these high-resolution structures with bound l-Arg analogues as well as the l-Arg-bound and l-Arg-absent structures, we note a very nice transition of the metal chelation, which is influenced by the position and orientation of the guanidine nitrogen of the ligand (Figure 3).

Combined with the changes at the C5 atom, these observations explain the reduced activity when l-Arg is replaced by l-canavanine. In particular, the orientation of l-canavanine with its O5 atom positioned away from the metal site leads to an inability of the first-shell residue D191 to bind the metal using OD2 that now appears to be critical for ethylene generation. Prior studies with the D191E variant of the EFE also emphasize the importance of the carboxylate metal ligand.^8^ The conservative alteration of D191 led to reduced activity, a smaller ratio of ethylene production versus l-Arg oxidation, and an increased concentration of the ferryl reaction intermediate. Structural studies of the D191E variant enzyme revealed two conformations for E191, one that mimics the orientation of D191 and one that significantly alters the coordination geometry around the Fe(II). Notably, the D191E EFE is the only structurally characterized variant thus far reported for this enzyme; below, we expand structural characterization to the R171A variant, eliminating a residue that stabilizes C1 of 2OG, and Y306A, a variant with an altered residue that lies distant from the active site in the WT EFE.^9^

### MD Simulations of the l-Canavanine-Bound EFE

**Figure 4:**
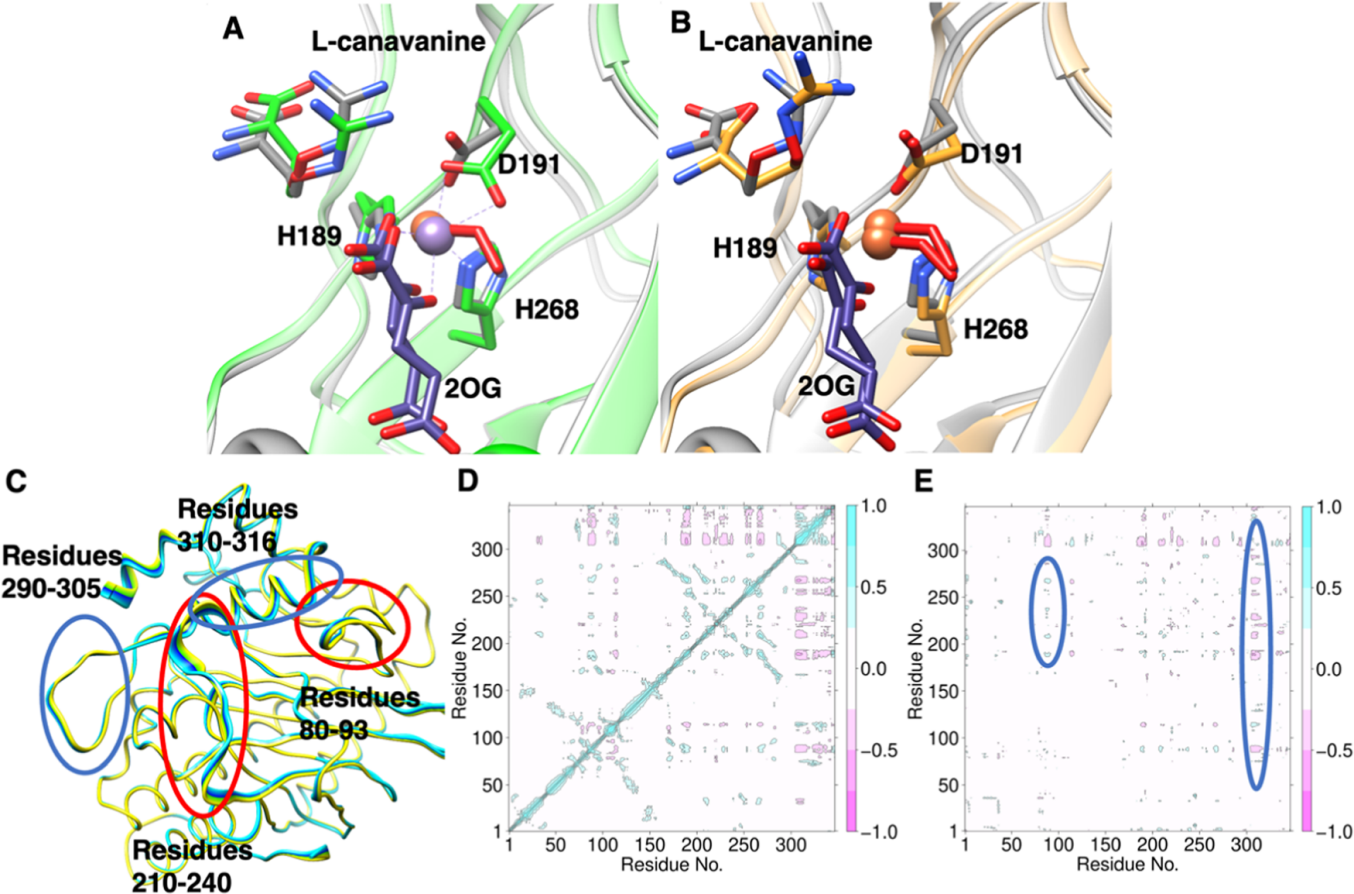
MD analysis of EFE/l-Can. (A) MD average snapshot of EFE·Fe(III)·OO^•^^–^·2OG·l-canavanine with l-canavanine bound in conformation B (gray) and the crystal structure of EFE·Mn·2OG·l-canavanine (green) (PDB: 6CBA). Fe(II) and Mn(II) are shown as orange and purple spheres, respectively, and the coordinated dioxygen is illustrated by red sticks. (B) MD average snapshots of EFE·Fe(III)·OO^•^^–^·2OG·l-canavanine comparing l-canavanine conformations A (orange) and B (gray). (C) PCA of the MD for EFE·Fe(III)·OO^•^^–^·2OG·l-canavanine: circled regions depict the parts of the enzyme that differ (blue) or show similarity (red) in flexibility compared to EFE·Fe(III)·OO^•^^–^·2OG·l-Arg (Figure S4). (D) DCCA showing the anticorrelated (pink) and correlated (blue) motions of EFE/l-Can. (E) d-DCCA, showing the loss of anticorrelation (blue) between β4 and β5 (positions 80–93) and the increased anticorrelation (pink) of β8 residues with other regions of the enzyme comparing EFE/l-Can versus EFE/l-Arg.

To explore the conformational flexibility of the EFE with bound l-canavanine, we performed MD simulations of the O_2_-bound EFE·Fe(III)·OO^•^^–^·2OG·l-canavanine complex (EFE/l-Can) (Figure S1).^5^ Our previous computational study explained the conformational motions of the EFE·Fe(III)·OO^•^^–^·2OG·l-Arg complex (EFE/l-Arg) in extensive detail.^5^ The present study reveals that as in EFE/l-Arg, the substrate analogue in EFE/l-Can exhibits two conformations: one with the O5 atom facing toward the metal (conformation A, Figure 4B) (46.3% of the 1 μs trajectory) and the other with O5 facing away from the metal (conformation B, Figure 4A,B) (53.6% of the 1 μs trajectory) (Figure S2). Although the population of conformation B in EFE/l-Can is reduced compared to that in the EFE/l-Arg system (11.7% conformation A and 88.3% conformation B),^5^ state B remains the most populated conformation, in agreement with the crystal structure. The key hydrogen bonds and salt bridge interactions between the C5 carboxylate of 2OG and R277 (92%) and between the C1 carboxylate of 2OG and R171 (70.4%) are stable through the MD simulations, consistent with the crystal structure of the EFE with l-canavanine (Figure 4A) and the MD simulations of EFE/l-Arg. In snapshots with l-canavanine conformation A, the guanidine group εN forms a stable hydrogen bond with nonbonded OD1 of D191 (46.3%), while such an interaction is less prominent in snapshots with l-canavanine in conformation B. Importantly, the oxygen atom at the fifth position of l-canavanine has van der Waals interactions with E84 in the case of conformation B (25%, with distances varying between 2.7 and 3.6, 3.3 Å between O5 of l-canavanine and the carboxylate oxygens of E84), similar to the crystal structure (Figure S3), which disappears in conformation A where the oxygen faces toward the metal, moving away from E84. The reason that bidentate D191 is observed in the crystal structure might be because l-canavanine in conformation B does not lead to a hydrogen bond between D191 and l-canavanine. The transition from intermediate bidentate-coordinated D191 to monodentate coordination might occur upon the binding of dioxygen.

PCA is a tool that helps analyze the motions of the flexible regions of proteins and discriminates relevant conformational changes.^47^ Compared to the PCA of EFE/l-Arg (Figure S4), the EFE/l-Can system had reduced flexibility (Figure 4C). The flexibility observed in residues 210–240 and 290–305 was substantially reduced. Unlike the EFE/l-Arg simulation, the EFE/l-Can system showed increased flexibility in the β8 strand (residues 310–316). This change of flexibility is reflected in the interdependent correlated motions as well. Based on the d-DCCA (Figure 4E) between simulations of the protein with l-Arg and l-canavanine, the simulations with l-canavanine showed a substantial loss of anticorrelated motion between the loop connecting β4 and β5 (80–93) and the β11 loop (210–240) and reduced anticorrelated motion between β4 and β5 (80–93) and the loop containing β15 and α8 (290–305). There is, however, an increased anticorrelated motion between β8 (residues 310–316) and the three loops mentioned above, which is not present in the case of l-Arg (Figure 4C). l-Canavanine exhibits correlated motion with β5 and anticorrelated motion with β8. The motion of these loops is important as they form the hydrophobic environment of the active site, which determines the substrate analogue and 2OG conformations. The DCCA of EFE/l-Can also revealed correlation between β15 and the loop connecting to α8, associated with active site hydrophobic residues including F283, F250, and A282 that interact with dioxygen (Figure 4D). The above residues exhibited anticorrelated motion in EFE/l-Arg. This combination of changes in the correlated motions of the EFE in the presence of the l-canavanine, in addition to the differences in the substrate analogue conformation and second-sphere hydrogen bonding interactions, could contribute to structural changes and ultimately to the loss of ethylene forming activity of the enzyme compared to the l-Arg-bound EFE. Overall, the results suggest the importance of the second coordination sphere and long range interactions in promoting EFE activity.

### Experimental Studies of the R171A Variant of the EFE

Anaerobic UV–vis spectroscopy demonstrated a lack of MLCT transitions when Fe(II) and 2OG were added to the R171A EFE, regardless of whether l-Arg was also provided (data not shown). This result suggested that either Fe(II) or 2OG fails to bind to the enzyme or they do not form the chelate structure that is typically associated with Fe(II)/2OG-dependent oxygenases.

**Figure 5:**
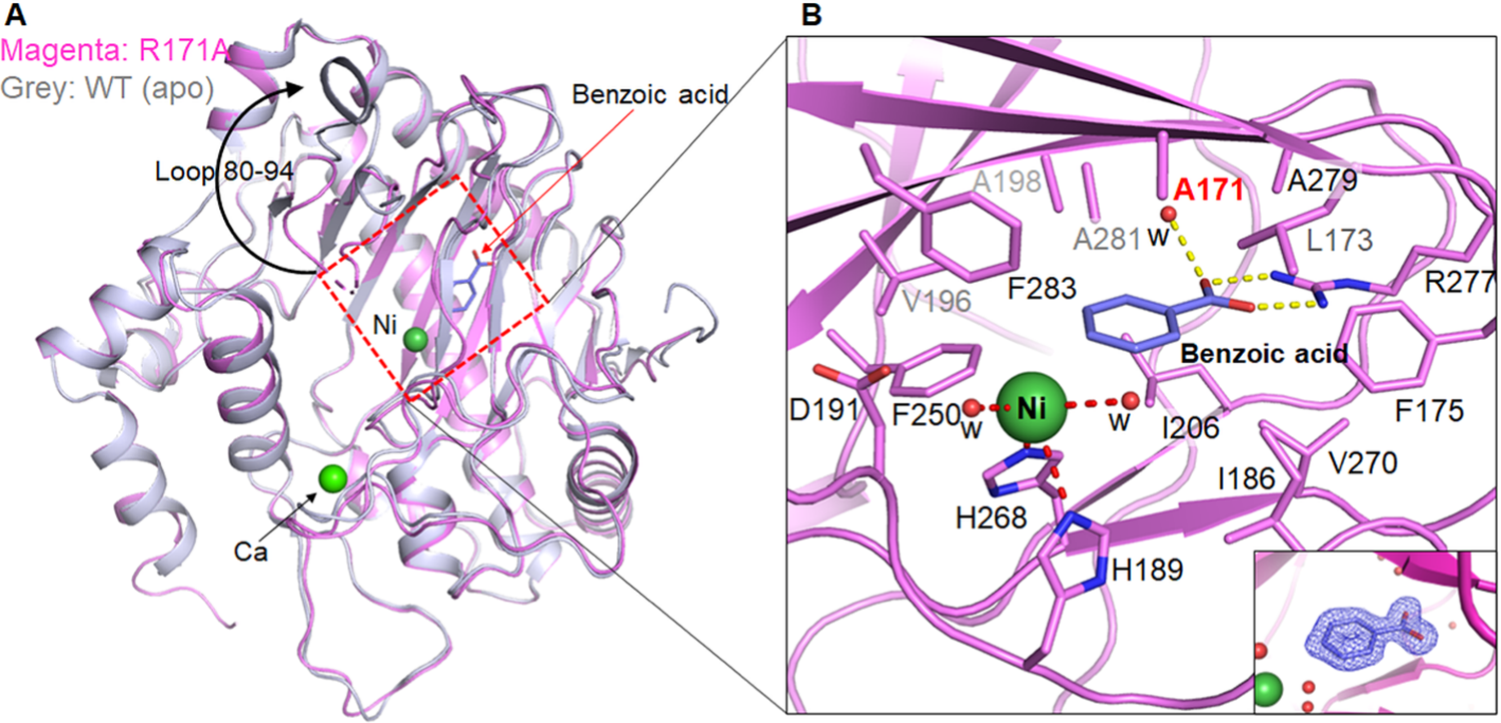
Structural comparison of the R171A variant (PDB: 8UC2) and WT EFE in the apoprotein (apo) state (PDB: 5V2U). (A) Structural superimposition of the two structures. A large conformational change of the loop containing residues 80–94 is indicated by the black arrow. (B) Close-up view of the active site of the R171 variant. An unexpected ligand, benzoic acid, was identified in the crystal structure. The 2Fo–Fc map (σ = 1) of benzoic acid is shown in the inset. The yellow dashed lines indicate the polar interactions of benzoic acid with R277 and a water molecule (w). The hydrophobic residues lining the 2OG binding site are shown and labeled. Ni(II) with 40% occupancy was identified at the metal binding site, and the red dashed lines indicate its coordination with H189, H268, and two water molecules.

We solved the structure of the presumed R171A variant apoprotein of the EFE to a resolution of 1.60 Å (PDB: 8UC2). Although no metal ions had been provided during crystallization, we observed electron density that was consistent with the bound metal at two sites (Figure 5A). The active site bound a metal at approximately 40% occupancy that was assigned to nickel, suspected to be derived during purification of the enzyme using the Ni-NTA resin. Ni(II) was bound to H189 and H268 but not to D191, which along with F250 exhibited distinct conformations when compared to other EFE structures. The second metal was bound to the backbone carbonyl atoms of N220, L222, and E225; this metal was tentatively assigned as Ca based on the electron density and geometry. The source of this metal ion is unknown, and it is not further discussed. Unexpectedly, electron density was present at the 2OG binding pocket, but this density did not match the structure of 2OG. Analysis of the crystals by LC-MS yielded a feature with the same mass and retention time (4.61 min) as benzoic acid (Figure S5), in agreement with the appearance of the electron density (Figure 5B, inset). By contrast, we eliminated nicotinic acid and salicylic acid as possible ligands based on their distinct elution positions (1.21 and 4.81 min, respectively) and masses. The carboxylate group of benzoic acid forms a salt bridge with R277, which normally forms a salt bridge with the C5 carboxylate of 2OG in the WT protein. Surrounding the aromatic ring of this molecule is a cage of hydrophobic residues: L173, I186, V196, A198, L206, F250, V270, A279, A281, and F283 (Figure 5B). The source of benzoic acid in the protein is unknown, but it must be obtained from the medium during growth of the recombinant cells or during variant enzyme isolation. Benzoic acid was found to not inhibit the WT EFE when tested at 5 mM concentration, indicating that it cannot outcompete 2OG binding even when at 10-fold excess. We postulate that the reason why this compound was able to bind to the R171A variant but not to the WT EFE when 2OG was not added in the crystallization buffer is that the deletion of the bulky and charged side chain of R171 leaves an open and hydrophobic space for this compound to enter (Figure 5B). Overall, these studies reveal that substitution of a substrate-binding, second-sphere residue can lead to profound changes at the active site.

### MD Simulations of the R171A EFE

**Figure 6:**
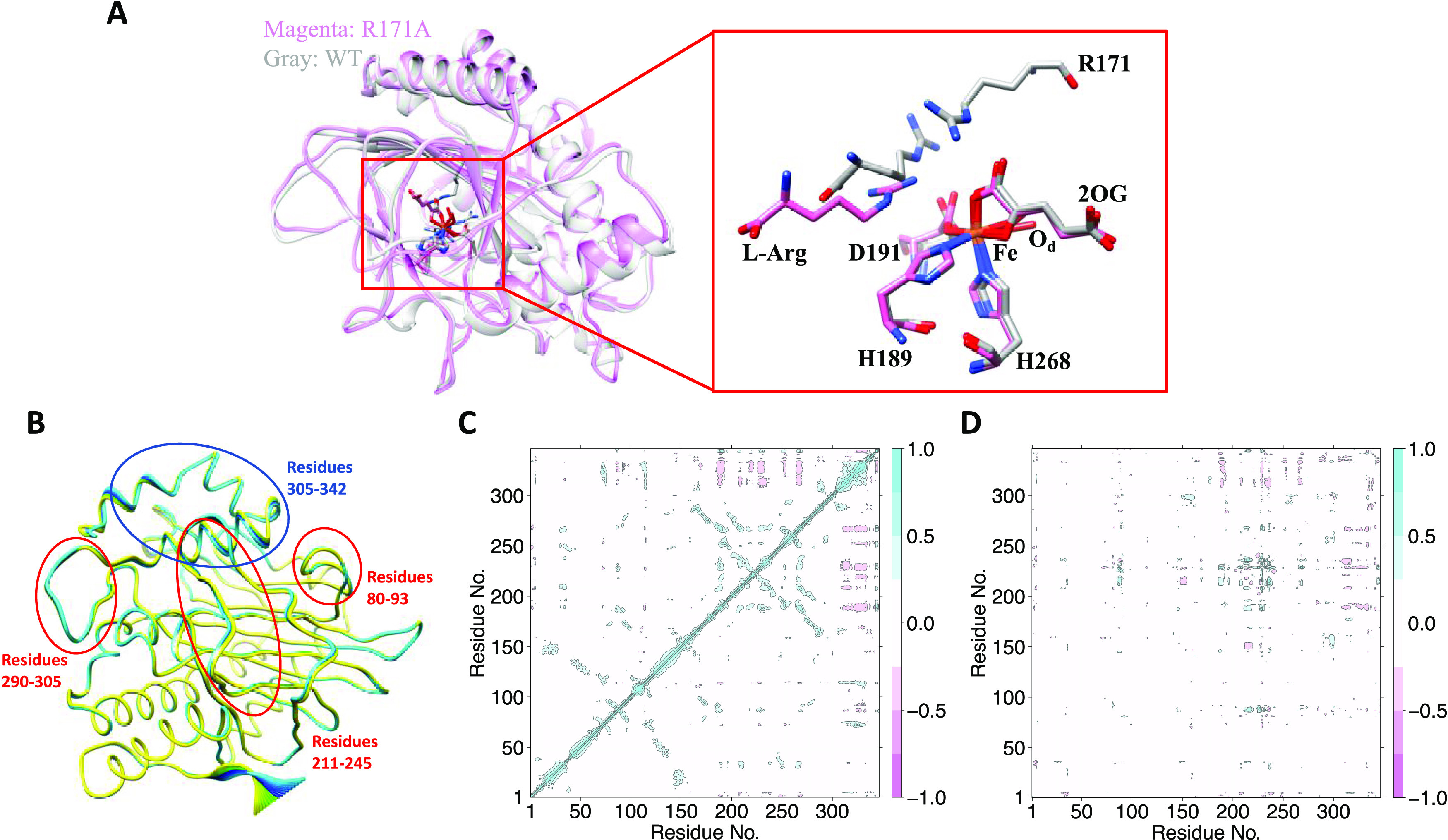
MD simulation of the R171A EFE. (A) Overlay of the average MD snapshots for the WT and R171A variant EFE. The active site residues are expanded for clarity on the right. The WT EFE is shown in gray, and the R171A variant is magenta. (B) PCA showing the flexible regions in the R171A EFE. (C) DCCA and (D) d-DCCA comparing the correlated motions in the WT and R171A EFE.

Prior experimental and computational studies of the WT EFE had revealed the role of R171 in stabilizing the off-line conformation of 2OG;^5,9^ i.e., an orientation with an oxygen-binding site that is not directed toward l-Arg. Further, R171 plays a decisive role in l-Arg positioning since the guanidinium group of R171 forms an electrostatic interaction with this substrate.^5^ Loss of these interactions might disrupt the substrate orientation in the active site. We performed a 1 μs MD simulation of the ferric-superoxo complex of the R171A variant to compare the substrate interactions to the WT enzyme (Figure 6A). The stability of the MD simulation was determined based on the RMSD of the Cα-carbons in the EFE variant (Figure S6). Previous studies had shown that in the WT EFE, η^2^ of the NH_2_ and εNH in l-Arg forms 44 and 68% hydrogen bond interactions with D191, respectively. Furthermore, E84 makes a hydrogen bond interaction with the ammonium group of the l-Arg in the WT EFE.^5^ Notably, substrate interactions with D191 and E84 are absent in the R171A variant, indicating substrate reorientation in the active site. Instead of E84, the backbone carbonyl of V85 interacts with the ammonium group of the l-Arg (45%), and the guanidinium group flips between the C1 carboxylate oxygens of the 2OG and D191 (Figure S7). The time-dependent fluctuations of the distance between Fe and the C5 hydrogen in the variant demonstrate the existence of both conformations A and B (Figure S8). At the same time, the position of the guanidinium group varies in the WT and variant enzymes (Figure 6A). The salt bridge interaction of R277 with the C5 carboxylate of 2OG is retained in the R171A variant (85%). These interactions illustrate the substitution’s impact on the enzyme’s active site. Additionally, MMGBSA calculations indicate weak l-Arg binding in the variant enzyme with a binding free energy of −17.0 kcal mol^–1^ in comparison to the −35.2 kcal mol^–1^ for the WT EFE.

PCA demonstrated the motion of the flexible regions in the R171A EFE (Figure 6B). Three regions in the WT enzyme were shown to be considerably flexible; however, the flexibility of the region involving residues 291–303 (a loop that connects with α8 and β15) is completely lost in the variant. The other flexible regions in the WT EFE involve (i) residues from 80 to 93 (a loop connecting β4 and β5) and (ii) a loop connecting β11 (residues 211–245). The flexibility of these regions in R171A was appreciably lost compared to the WT enzyme; these changes might affect the binding of 2OG and l-Arg. Strikingly, regions of α7, α8, α9, and connecting residues (positions 305–342) in the R171A EFE were shown to move away from the active site. There was also a slight increase in the flexibility of the loop connecting β8 and β9 (residues 189–195) in the variant enzyme compared to the WT EFE. Thus, the PCA results reveal substantial changes in the motion of the flexible region as an implication of the substitution of R171.

DCCA elucidates the differences in correlated motions between the R171A and WT EFE (Figure 6C,D). Previous studies have shown that R171 positively correlated with the residues of β6 and β14.^5^ The variant showed no remarkable correlation with β6 and β14 but demonstrated anticorrelated motions with residues of α8 and α9; the loss of positive correlation might affect the l-Arg orientation in the active site. Furthermore, the correlated motion of the region encompassing the loop connecting α8 and β15, along with the loop connecting β4 and β5, and the loop linking β11, which played a crucial role in l-Arg binding in the WT EFE, is disrupted in the variant. The loss of these correlated motions with the loop connecting α8 and β15 might have destabilized l-Arg binding in the active site. The region consisting of residues 305–342 (α7, α8, α9, and its connecting residues) in the variant makes an asynchronous (anticorrelated) motion with the regions connecting β4 and β5, β8 and β9, and the loop joining β11. Hydrophobic residues (F250, A281, and F283) interacting with active site residues showed no anticorrelated motion in the variant enzyme, as seen in the WT enzyme. The increased flexibility and correlated motions of the region constituting residues 305–342 might be due to the loss of interaction of R171 with the residues of α7, which in turn might affect the l-Arg stability in the active site. The MD study of the R171A EFE reveals a significant alteration in the l-Arg conformation and long-range correlated motion, potentially contributing to the reduced catalytic efficiency of this variant.

### Experimental Studies of the Y306A Variant of the EFE

**Figure 7:**
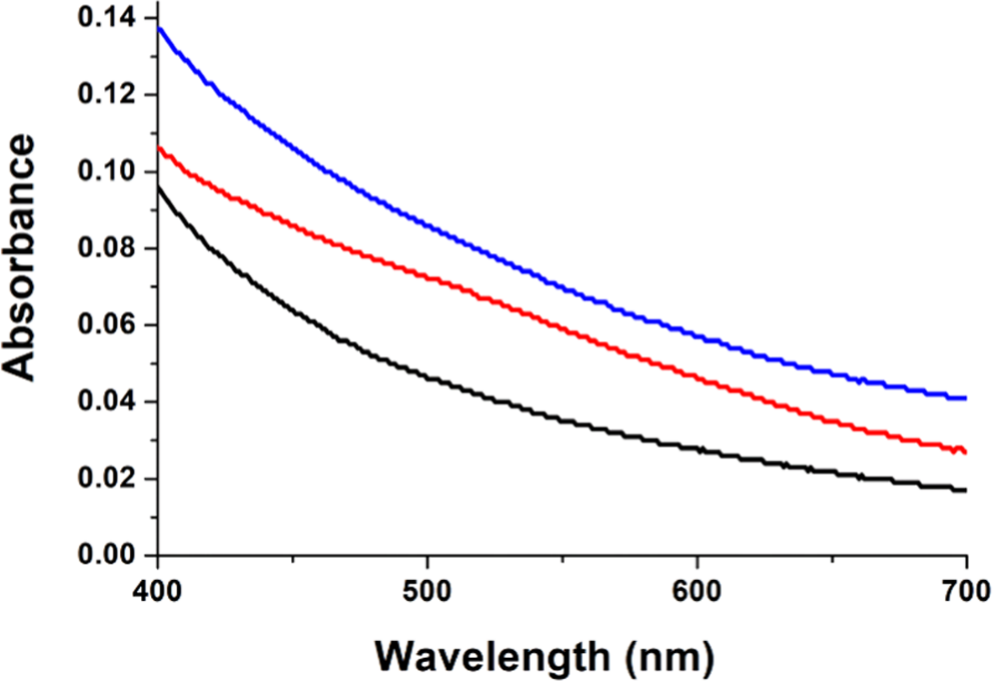
Anaerobic UV–vis spectra of Y306A EFE·Fe(II) (black), Y306A EFE·Fe(II)·2OG (red), and Y306A EFE·Fe(II)·2OG·l-Arg (blue).

Y306 is a conserved residue that we proposed to be important because of its hydrophobic interactions.^9^ Compared to the WT enzyme, Y306A and Y306F possess ethylene-forming activities of 4 and 6% and have P5C-forming activities of 5 and 10%, respectively. The addition of 2OG to Y306A EFE·Fe(II) led to a very small increase in absorption at ∼525 nm (calculated to be ∼130 M^–1^ cm^–1^), consistent with only partial chelation of Fe(II) by 2OG. For WT EFE·Fe(II)·2OG, the addition of l-Arg or l-canavanine led to an increase in these MLCT transitions (Figure 1); however, l-Arg addition to Y306A EFE·Fe(II)·2OG appeared to eliminate this absorption peak and generated an increase in light scattering (Figure 7). We conclude that the Y306A variant of the EFE does not bind l-Arg in the same manner as seen for the WT enzyme and fails to promote bidentate binding of 2OG.

**Figure 8:**
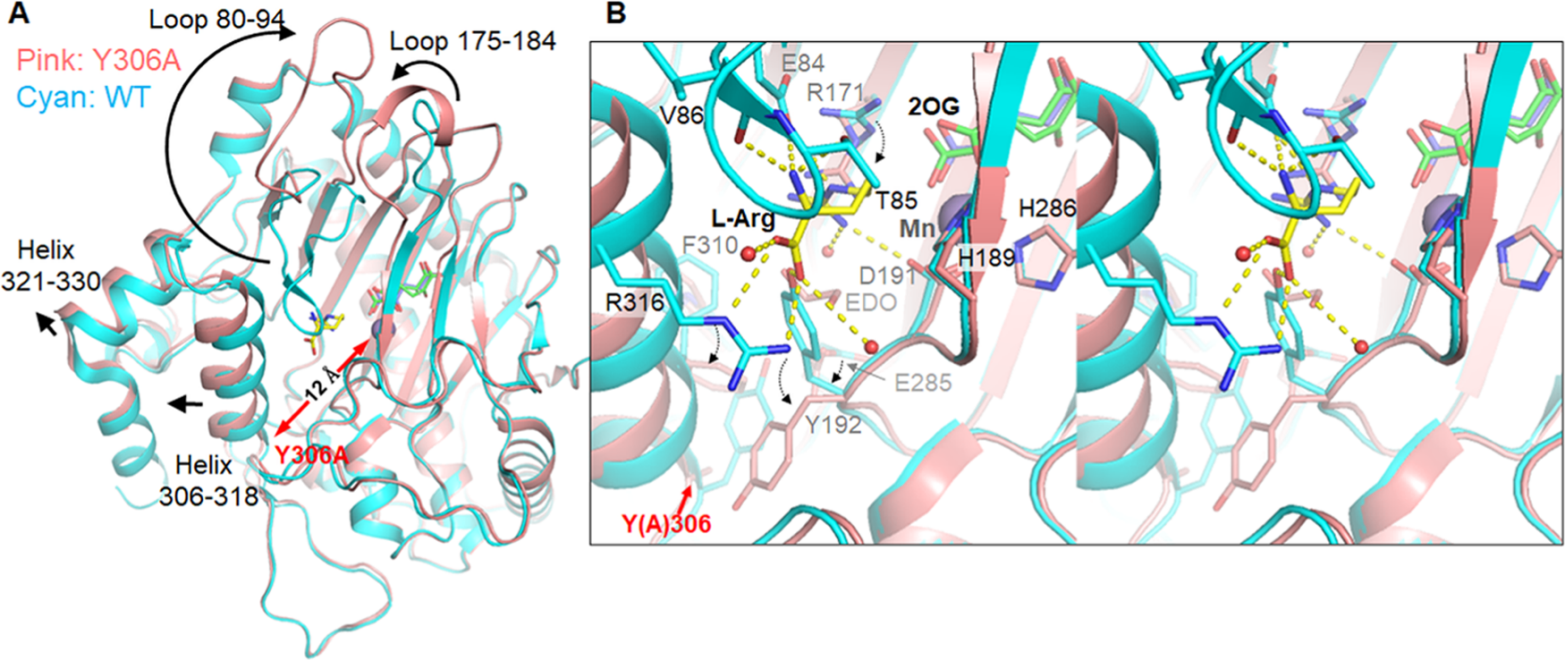
Structural comparison of the Y306A variant EFE (PDB: 6CF3) and WT EFE (PDB: 5V2Y). (A) Structural superimposition of the Y306A EFE·Mn(II)·2OG complex and the EFE·Mn(II)·2OG·l-Arg complex. Significant conformational changes were identified for four secondary structural elements, which are indicated by the black arrows. (B) Stereo-view of the active sites of the WT and the Y306A variant EFE. The black curved arrows indicate the significant structural changes of four residues of the Y306A variant when compared to the corresponding residues in the WT EFE. The yellow dashed lines indicate the polar interactions of l-Arg (sticks with yellow carbon atoms) with the residues in the l-Arg-binding site and water molecules. An ethylene glycol (EDO) molecule was identified in the Y306A structure.

The Y306A variant of EFE was cocrystallized with MnCl_2_, 2OG, and l-Arg, but the structure solved at 1.12 Å (PDB: 6CF3) showed a Y306A EFE·Mn(II)·2OG complex, lacking l-Arg, which is in contrast to all WT EFE structures cocrystallized with similar compounds. As a result, the Y306A EFE·Mn(II)·2OG complex exhibits structural features that are similar to those of the EFE·Mn(II)·2OG complex (PDB: 5V2X). In particular, the 2OG adopts two conformations—it is coordinated in a bidentate manner with the metal in a productive conformation (with an occupancy of 63%) or in a monodentate manner with the metal in an opposite orientation (with an occupancy of 37%); the structural observation of this mixture matches results from the UV–vis studies. The latter conformation is observed only when l-Arg is absent in the crystal structure for the WT EFE. When compared to the l-Arg-bound structure of the WT EFE, several structural elements are in different conformations (Figure 8A). These include the loop containing residues 80–94, which form multiple hydrogen bonds with l-Arg in the WT enzyme but flip away from the l-Arg-binding site in the variant structure; the loop containing residues 177–184, which fold into a one-turn helix not seen in the l-Arg-bound structure; and the helix containing residues 306–318 and 321–330 that shift away from the l-Arg binding site.

The close-up view of the superimposed structures reveals the structural basis of the loss of l-Arg binding capacity for the Y306A variant of the EFE (Figure 8B). Substitution of the bulky Y306 by alanine leads to a cavity in the structure, which is occupied by F310 and Y192 that flip down their side chains from the positions in the l-Arg-bound state. The conformational change of Y192 appears to have a profound effect on l-Arg binding because it not only forms a hydrogen bond with the carboxylic acid group of l-Arg but also defines the shape of the l-Arg binding pocket and limits the flexibility of the bound ligand. The empty space created by the flipping of Y192 is only partially occupied by an ethylene glycol molecule (from the cryoprotectant), which is very unlikely to be able to compensate for the loss of the bulky and rigid side chain of Y192. The defect in l-Arg binding is further exaggerated by the loss of additional key interactions, including R316 in the helix associated with residues 306–318, which is shifted away from the l-Arg-binding pocket, and residues 84–86 in the loop involving positions 80–94 that flips away from the l-Arg-binding site. We and others have shown by substitution of the l-Arg-binding residues E84 and T86 that residues in this loop are crucial for ethylene formation.^9,10^ E84 is particularly important in that it not only forms hydrogen bonds with the amino and guanidine groups of l-Arg but also plays a role in orienting R171 via a salt bridge. R171 is a key residue in 2OG binding, and the reoriented R171 likely accounts for the nonproductive binding of 2OG. In the Y306A EFE structure, R171 adopts a conformation that would clash with the guanidine group of l-Arg if the latter was bound in the same way as in the WT enzyme. In addition, an altered orientation of the side chain of E285, which directly interacts with Y306, may also contribute to the inability of the variant to bind l-Arg, as this residue is connected to the ligand-binding site via ordered water molecules. Taken together, this study demonstrates a case where an interior cavity created by a single amino acid substitution 12 Å away from the catalytic metal center leads to local and distal structural changes that significantly reduce the enzyme’s activity.

### MD Simulations of the Y306A EFE

We explored the conformational flexibility of the Y306A variant of the EFE in the presence and absence of the l-Arg substrate. A 1 μs MD simulation was performed utilizing the crystal structure of the Y306A EFE to explore the conformational changes in the absence of l-Arg (Figure S9). Like what was found in the crystal structure, a salt bridge interaction between R277 and the C5 carboxylate oxygen and a hydrogen bonding interaction between R171 and the nonbonding carboxylate oxygen stabilize the binding of 2OG. The MD simulation showed that the side chains of F310 and Y192 occupy the vacancy created by the substitution of Y306, as in the crystal structure. However, these residues are dynamic with high flexibility and occupy different orientations (Figure S10); hence, they may not contribute to the l-Arg binding as effectively as in the WT EFE. Similarly, the side chain of R171 is flexible and can influence the l-Arg binding (Figure S11). The loop 80–94 and helical residues 306–318, which move away from the active site in the crystal structure, are also highly flexible in the MD (Figure S9B). The combined effect of the flexibility of the loop and helical regions, along with the openness of the side chains, potentially destabilizes l-Arg binding in the active site, which might have prevented the crystallization of the substrate.

**Figure 9:**
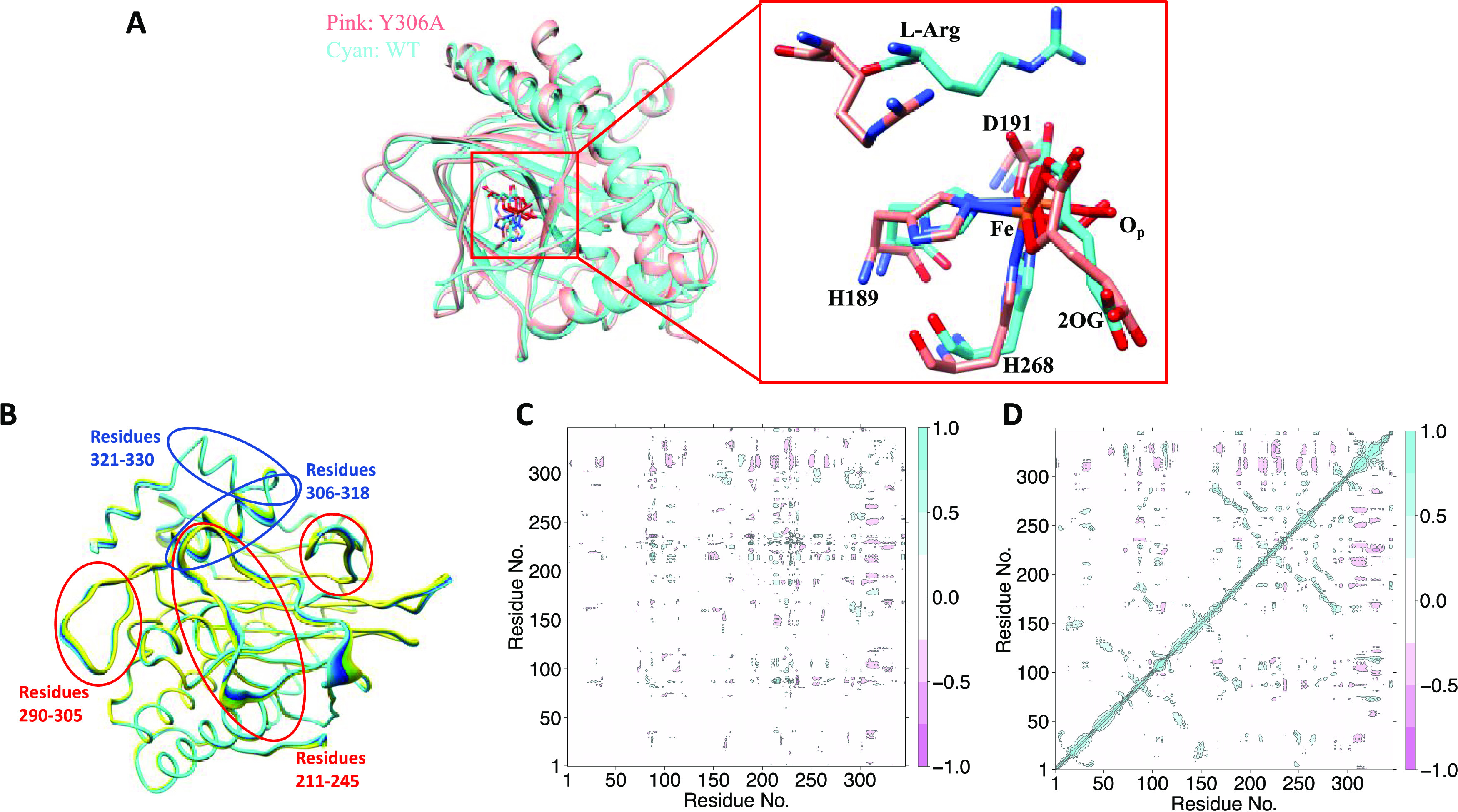
MD simulation of Y306A EFE·Fe(III)·OO^•^^–^·2OG·l-Arg. (A) Overlay of MD snapshots of the WT and Y306A variant EFE. The active site residues are expanded on the right. The WT EFE is shown in cyan, and the variant is shown in pink. (B) PCA showing the flexible regions in Y306A. The regions in red are flexible in the WT and variant enzymes, whereas the region in blue shows flexibility only in the variant EFE. (C) d-DCCA and (D) DCCA comparing the correlated motions in the Y306A and WT EFE.

The Y306A variant exhibits very low rates of ethylene production and l-Arg hydroxylation (i.e., 3–5% of that for the WT enzyme), but it still has slight activity. To further understand how the Y306A substitution influences l-Arg binding, we performed a 1 μs MD simulation of Y306A EFE·Fe(III)·OO^•^^–^·2OG·l-Arg that was generated from the WT EFE crystal structure with bound 2OG and l-Arg after performing in silico substitution of Y306 by alanine (Figure S12). The simulation demonstrates substantial changes in the orientation of the substrate in the active site (Figure 9A) with respect to the WT EFE/l-Arg.^5^ The hydrogen bonding interaction of the C1 carboxyl groups of 2OG with R171 (47%) is weakened, and the salt bridge interaction of R277 with the C5 oxygen atoms is retained in the simulation for the variant enzyme. The guanidium group of l-Arg also makes weaker electrostatic interactions with R171 (Figure S13). Previous simulation of the WT EFE/l-Arg revealed that stronger interactions between the guanidium group of the l-Arg and R171 favor conformation A, while weaker interactions favor conformation B.^5^ Therefore, in the Y306A variant, l-Arg is displaced to conformation B (Figure S14), but the guanidinium group of l-Arg moves further away than in WT EFE/l-Arg as shown in Figure S13. The larger shift in the guanidinium group of l-Arg in the Y306A variant protein might be a factor in the low reactivity of this variant enzyme. The interaction of E84 with the amino group of l-Arg stabilizes conformation B in the WT EFE/l-Arg;^5^ this interaction is not present in Y306A, whereas the amino group interacts with the backbone of V85 (66%) (Figure S15). Further, the carboxylate group of the l-Arg does not interact with Y192 as in the WT enzyme since it is flexible due to the alanine substitution. Overall, the MD analysis shows that l-Arg exhibits weaker interactions with the residues in the variant protein compared to the WT EFE. Consistently, the calculated binding energy of the substrate using MMGBSA displayed weaker binding of l-Arg in the Y306A variant (−17.1 kcal mol^–1^) than in the WT enzyme (−35.2 kcal mol^–1^).

PCA showed that helix-forming residues 306–318 and 321–330 have high flexibility in the Y306A variant EFE, which was not observed in the WT enzyme (Figure 9B). Crystallographic studies have also demonstrated that this helical region shifts away from the l-Arg-binding site; therefore, the flexibility of these helical regions might destabilize binding of the substrate in the active site. d-DCCA of the WT and variant enzymes shows anticorrelated motion between the loop connecting β15 and α8 (residues 290–305) with the loop connecting β4 and β5 (80–93) and β11 loop (210–240). The lack of l-Arg in the Y306A EFE crystal structure is consistent with a significant decrease in binding affinity compared to the WT enzyme, implying that the long-range motions have a role in substrate binding (Figure 9C). Also, helical residues 306–318 and 321–330 show anticorrelated motions with the remaining regions of the protein, further indicating the influence of long-range correlations in stabilizing l-Arg binding (Figure 9D). The different correlated motions and binding interactions in the variant and WT EFE might reflect the destabilization of the substrate, thereby reducing the catalytic turnover of the Y306A EFE.

## Conclusions

l-Canavanine binds to the EFE with its O5 atom pointing away from the metal center, whereas C5 of l-Arg points toward the iron atom, consistent with the ability of the enzyme to react with only the latter compound. Congruent with the structural results, MD simulations predict that the most favored l-canavanine conformation in the EFE·Fe(III)·OO^•^^–^·2OG·l-canavanine complex has its O5 pointed away from the metal center and facing toward E84. Anaerobic UV–vis spectroscopy of EFE·Fe(II)·2OG·l-canavanine and structural studies of EFE·Mn(II)·2OG·l-canavanine confirm that binding of the substrate analogue leads to bidentate coordination of 2OG to Fe(II). Critical insights into why the l-canavanine-bound enzyme is ineffective for ethylene generation were obtained from the crystal structure of EFE·Mn(II)·2OG·l-canavanine that revealed that OD1 of D191 coordinated the metal instead of the catalytically productive OD2 coordination noted for bound l-Arg.

Alanine substitution of R171, a residue that forms a hydrogen bond with the C1 carboxylate of 2OG, leads to a significant shift of the loop containing residues 80–94, a loss in activity, and an inability to form the chromophore associated with chelate binding of Fe(II) by 2OG. These changes cause reduced stability of binding 2OG, leading to the adventitious binding of benzoate at the 2OG binding site. The source of benzoate is unclear, analogous to the situation for crystals of canavalin, a jack bean vicilin protein, that was purified from *E. coli* cells and shown to have bound benzoate.^48^ Both the UV–vis studies and MD simulations suggest a weakened binding of l-Arg for this variant protein.

Y306 is a generally conserved residue located 12 Å from the metal site, with the active site residue Y192 located in between. We had proposed that Y306 was important because of its hydrophobic interactions and its role in forming a hydrogen bond between its hydroxyl group and E285.^9^ Substitution of E285 to alanine or glutamine abolished ethylene formation while maintaining 15 or 20% of P5C formation, respectively. The Y306 and E285 side chain orientations were the same in all WT enzyme structures, regardless of the presence of metal, 2OG, or l-Arg. However, E285 also forms hydrogen bonds to water molecules that connect to the backbone of D191 and Y192. A water-to-water hydrogen bond links E285 to the guanidine group of l-Arg and l-Arg analogues as well as to H169. These results indicate that there is a connection of Y306 to the active site that could explain the reduction in activity for the Y306A variant. The structure of this protein now offers an even more clear and surprising picture of major changes introduced by a single-point substitution. Substitution of Y306 by alanine leads to a large conformational change of one loop with additional propagated changes to affect several residues important for l-Arg binding, accounting for the inactivity of this variant. UV–vis and structural studies both reveal that 2OG binds to the protein to yield a mixture of the monodentate- and bidentate-coordinated species. The loss of the MLCT transitions upon addition of l-Arg is consistent with a deficiency in l-Arg binding as revealed by its absence of this amino acid in the crystal structure despite being present in the crystallization buffer. In line with experimental studies, the binding energy calculated from the MD simulations using MMGBSA revealed a lower binding affinity of l-Arg in the Y306A variant enzyme.

In combination, the results presented here are compatible with the currently proposed enzyme mechanism,^4−8^ emphasize the necessity for OD2 of D191 to coordinate the metal, and highlight the importance of the second coordination sphere and longer range interactions in promoting EFE activity. Such interactions must be kept in mind when investigating variant forms of the EFE for enhanced production of ethylene as a biofuel or for making increased amounts of guanidine as an agricultural fertilizer using recombinant microorganisms.^49−53^

## Supporting Information Available

Crystal statistics for structures of 6CBA, 6CF3, and 8UC2, MD analysis for the three samples along with conformation analyses, predicted hydrogen bonding interactions, and PCA, and ESI-MS analysis of the organic compound extracted from R171A crystals (PDF)

## Accession Codes

Uniprot accession code for EFE: P32021

## Dedication

This article is dedicated to the memory of Christopher T. Walsh, an outstanding mechanistic enzymologist and postdoctoral mentor to RPH.
