# Supplementary material for: Structural, Spectroscopic, and Computational Insights from Canavanine-Bound and Two Catalytically Compromised Variants of the Ethylene-Forming Enzyme
Source: Biochemistry. 2024 Apr 5;63(8):1038–50. doi: 10.1021/acs.biochem.4c00031 (PMC11025135; doi:10.1021/acs.biochem.4c00031)
Supplement: Supplementary file 1 — bi4c00031_si_001.pdf [file bi4c00031_si_001.pdf]

Supporting Information

Structural, Spectroscopic, and Computational Insights from Canavanine-Bound and Two Catalytically Compromised Variants of the Ethylene-Forming Enzyme

Shramana Chatterjee,[1,‖] Matthias Fellner,[2,3,‖] Joel A. Rankin,[1,2,4] Midhun G. Thomas,[5] Simahudeen Bathir J. S. Rifayee,[5] Christo Z. Christov,[5,*] Jian Hu,[2,6,*] and Robert P. Hausinger[1,2,*]

[1]Department of Microbiology, Genetics, and Immunology, Michigan State University, East Lansing, MI 48824, USA
[2]Department of Biochemistry and Molecular Biology, Michigan State University, East Lansing, MI 48824, USA
[3]Present address: Biochemistry Department, School of Biomedical Sciences, University of Otego, Dunedin 9054, New Zealand
[4]Present address: Department of Biochemistry Molecular Biology and Biophysics, University of Minnesota, Minneapolis, Minnesota, 55108, USA
[5]Department of Chemistry, Michigan Technological University, Houghton, Michigan, 49931, USA
[6]Department of Chemistry, Michigan State University, East Lansing, MI 48824, USA

[‖]These authors contributed equally to this work

*Corresponding authors email addresses: christov@mtu.edu, hujian1@msu.edu, and hausinge@msu.edu.

Table S1. Crystal statistics for WT EFE·Mn·2OG·L-canavanine, Y306A variant EFE·Mn·2OG, and R171A variant EFE·Ni·benzoate.

**EFE crystals**	**WT EFE·Mn·2OG·L-canavanine**	**Y306A EFE·Mn·2OG**	**R171A EFE·Ni·benzoate**
**Data collection**			
Beamline	LS-CAT 21-ID-D	LS-CAT 21-ID-D	LS-CAT 21-ID-D
Wavelength (Å)	0.976	0.976	0.976
Detector distance (mm)	100	100	100
Space group	$P2_12_12_1$	$P2\ 2_12_1$	I 2 2 2
Unit cell a, b, c (Å)<br>α, β, γ (°)	48, 82, 87<br>90, 90, 90	44, 85, 87<br>90, 90, 90	80, 97, 98<br>90, 90, 90
[a]Resolution (Å)	48.43 – 1.13<br>(1.15 – 1.13)	44.37 – 1.12<br>(1.14 – 1.12)	62.03 - 1.60<br>(1.63 - 1.60)
Unique reflections	128,283 (6,221)	125,336 (5,884)	47283 (1380)
[a]Redundancy	7.2 (6.5)	5.9 (4.6)	5.8 (1.5)
[a]Completeness (%)	98.5 (97.5)	98.2 (94.0)	93.0 (56.1)
[a]$I/\sigma I$	11.3 (2.3)	7.9 (2.2)	20.0 (2.5)
[a,b]R_{merge}	0.075 (0.804)	0.111 (0.607)	0.045 (0.205)
[a,c]R_{pim}	0.044 (0.494)	0.072 (0.430)	0.027 (0.196)
[d]$CC_{1/2}$	0.999 (0.804)	0.992 (0.707)	0.999 (0.906)
**Refinement**			
Protein atoms	5,757	5,341	2747
Manganese atom	1	1	0
Nickel atom	0	0	1
Calcium atom	0	0	1
2OG molecule	1	1	0
L-Canavanine molecule	1	0	0
Benzoic acid molecule	0	0	1
Ethylene glycol molecule	0	1	2
H_2O molecules	470	351	464
[e]R_{work}/R_{free}	0.142 / 0.163	0.148 / 0.163	0.169/0.186
B-factors (Å^2)	16.3	14.2	23.2
Protein atoms	15.4	13.5	21.6
Manganese atom	9.7	7.8	-
Nickel atom	0	0	37.9
Calcium atom	0	0	41.9
2OG molecule	14.9	15.2	-
Benzoic acid molecule	0	0	14.8
L-Canavanine molecule	18.5	-	-
Ethylene glycol molecule	-	18.1	40.1
H_2O molecules	27.5	25.0	32.5
R.m.s. deviation in bond lengths (Å)	0.007	0.007	0.007
R.m.s. deviation in bond angles (°)	0.996	0.956	0.914
Ramachandran plot (%) favored	97.98	98.79	98.79
Ramachandran plot (%) outliers	0.58	0.30	0.30
Rotamer outliers (%)	0	0	0.69
PDB ID	6CBA	6CF3	8UC2

[a]Highest resolution shell is shown in parentheses.

[b]$R_{merge} = \sum_{hkl} \sum_j |I_j(hkl) - \langle I(hkl)\rangle| / \sum_{hkl} \sum_j I_j(hkl)$, where I is the intensity of reflection.

[c] $R_{pim}=\sum_{hkl} [1/(N-1)]^{1/2} \sum_j |I_j(hkl)-<I(hkl)>| / \sum_{hkl} \sum_j I_j(hkl)$, where N is the redundancy of the dataset.
[d] $CC_{1/2}$ is the correlation coefficient of the half datasets.
[e] $R_{work} = \sum_{hkl} | |F_{obs}| - |F_{calc}| | / \sum_{hkl} |F_{obs}|$, where F_{obs} and F_{calc} is the observed and the calculated structure factor, respectively. R_{free} is the cross-validation R factor for the test set of reflections (5% of the total) omitted in model refinement.

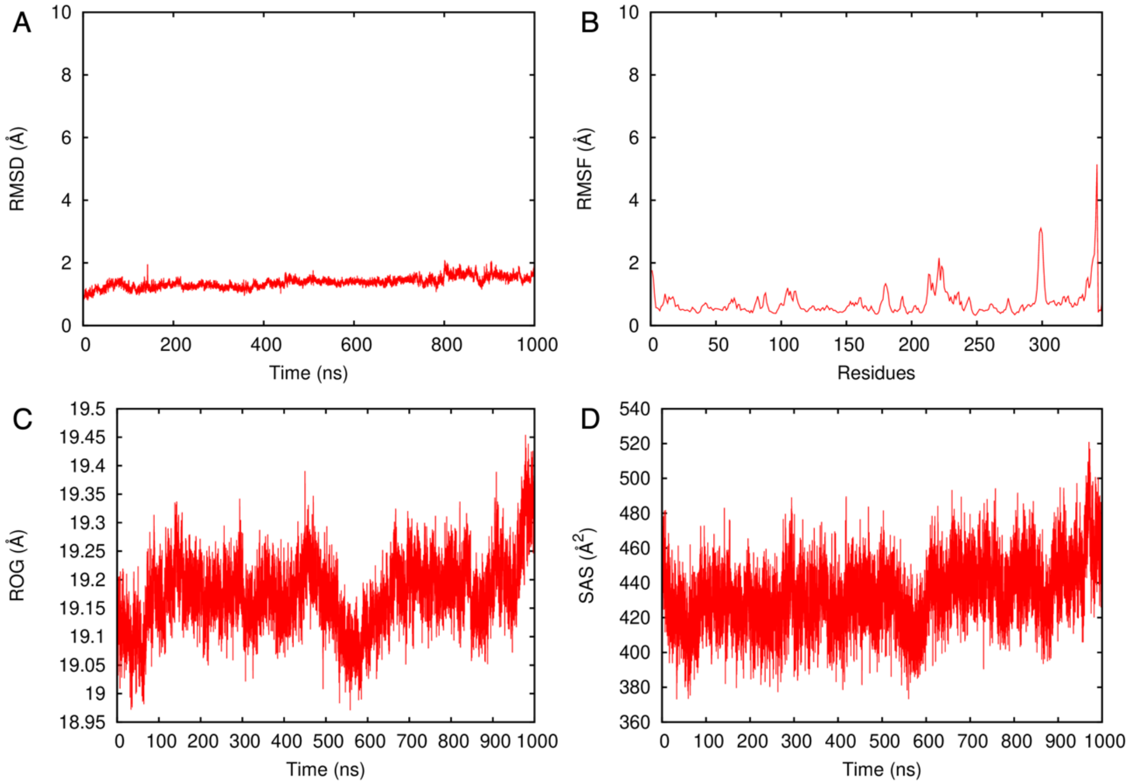

Figure S1. MD analysis of the L-canavanine substrate analog interacting with WT EFE. A) Root mean square deviation (RMSD) of the α carbons suggests the system is equilibrated. B) Root mean square fluctuation (RMSF) indicates the flexible regions of the protein. C) Radius of gyration (ROG) shows the stability of the overall protein fold. D) Solvent-accessible area (SAA) showing evidence of an equilibrated system.

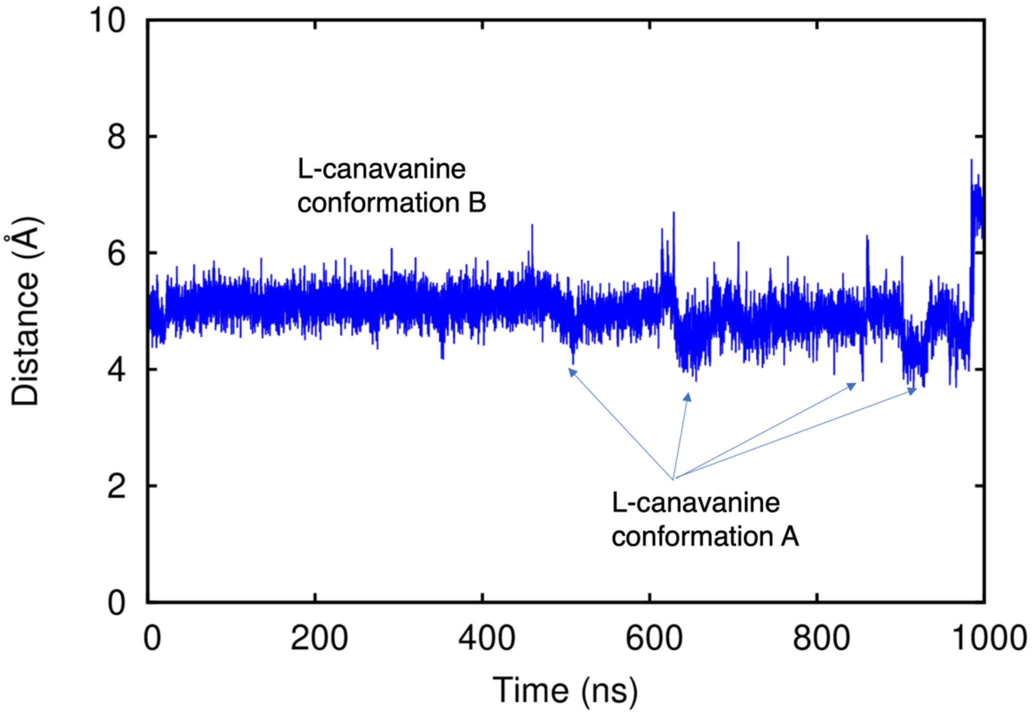

Figure S2. Plot depicting the change in distance between the EFE Fe and oxygen at the fifth position of L-canavanine during the dynamics shows the presence of A and B conformations of the substrate analog.

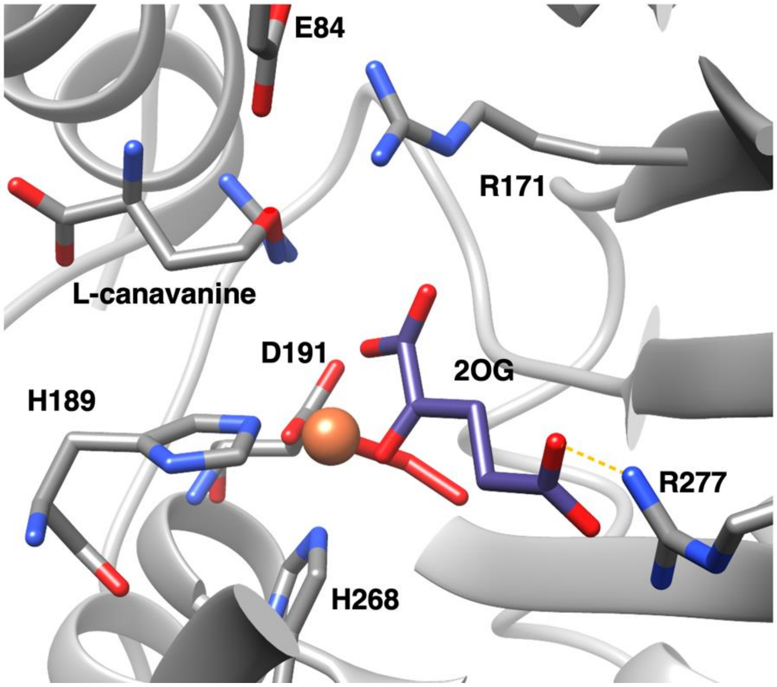

Figure S3. Representation of the van der Waals and hydrogen bonding interactions stabilizing the L-canavanine substrate analog in conformation B and 2OG in EFE/L-Can MD simulations.

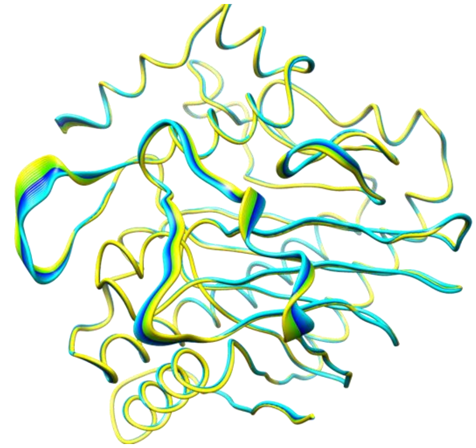

Figure S4. PCA of the MD for WT EFE/L-Arg depicting the flexibility in the protein.

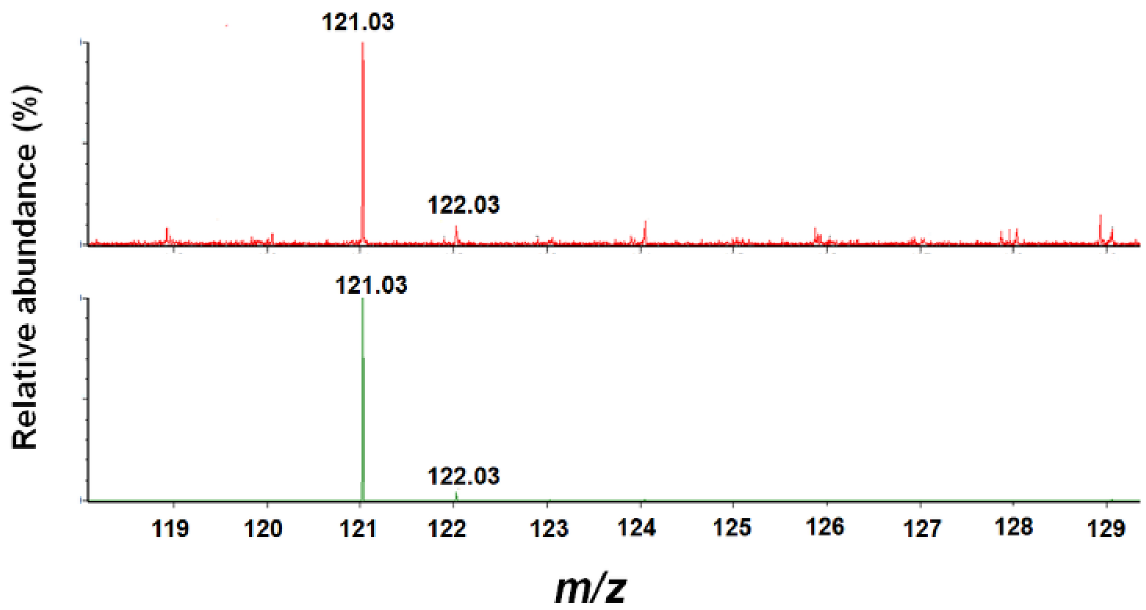

Figure S5. ESI-MS in negative ion mode of the sample extracted from the R171A EFE crystals (top) and a benzoic acid standard.

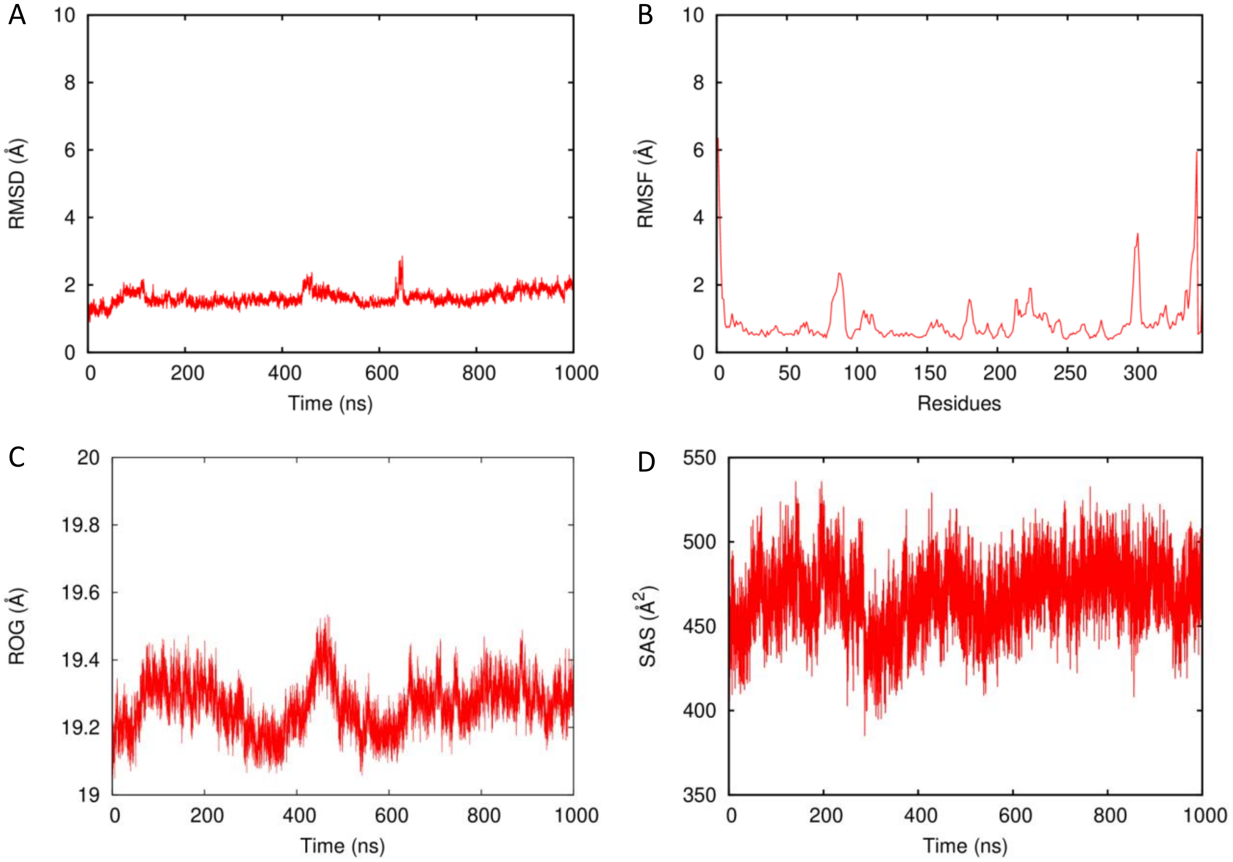

Figure S6. MD analysis of the R171A variant of EFE. A) RMSD of the α carbons suggests the system is equilibrated. B) RMSF indicates the flexible regions of the protein. C) ROG shows the stability of the overall protein fold. D) SAA showing evidence of an equilibrated system.

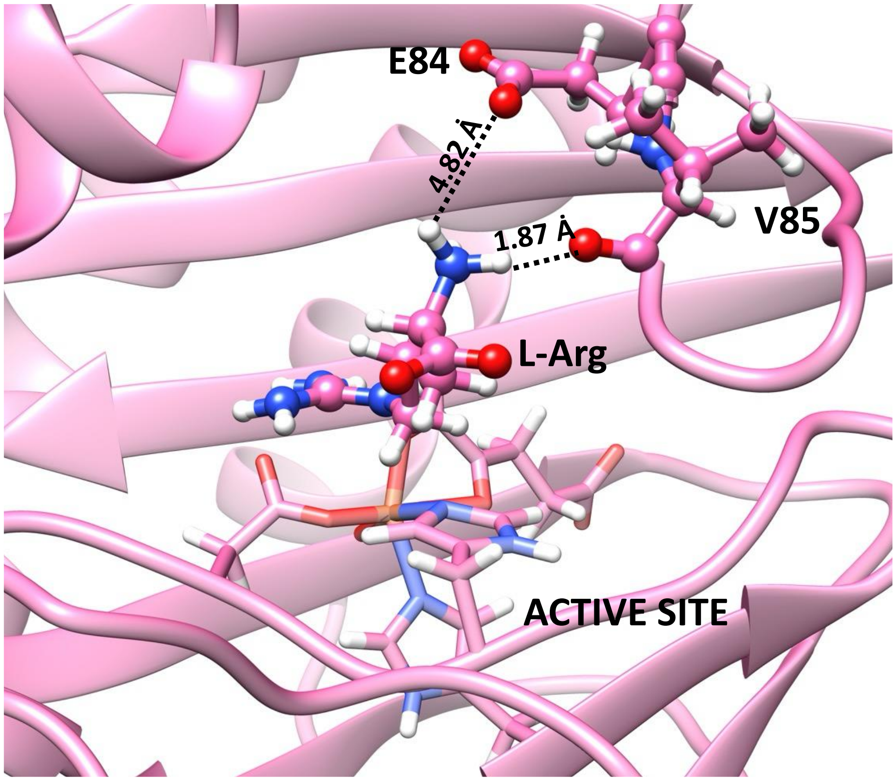

Figure S7. Representation of the hydrogen bonding interactions that stabilize L-Arg binding to the R171A variant of EFE.

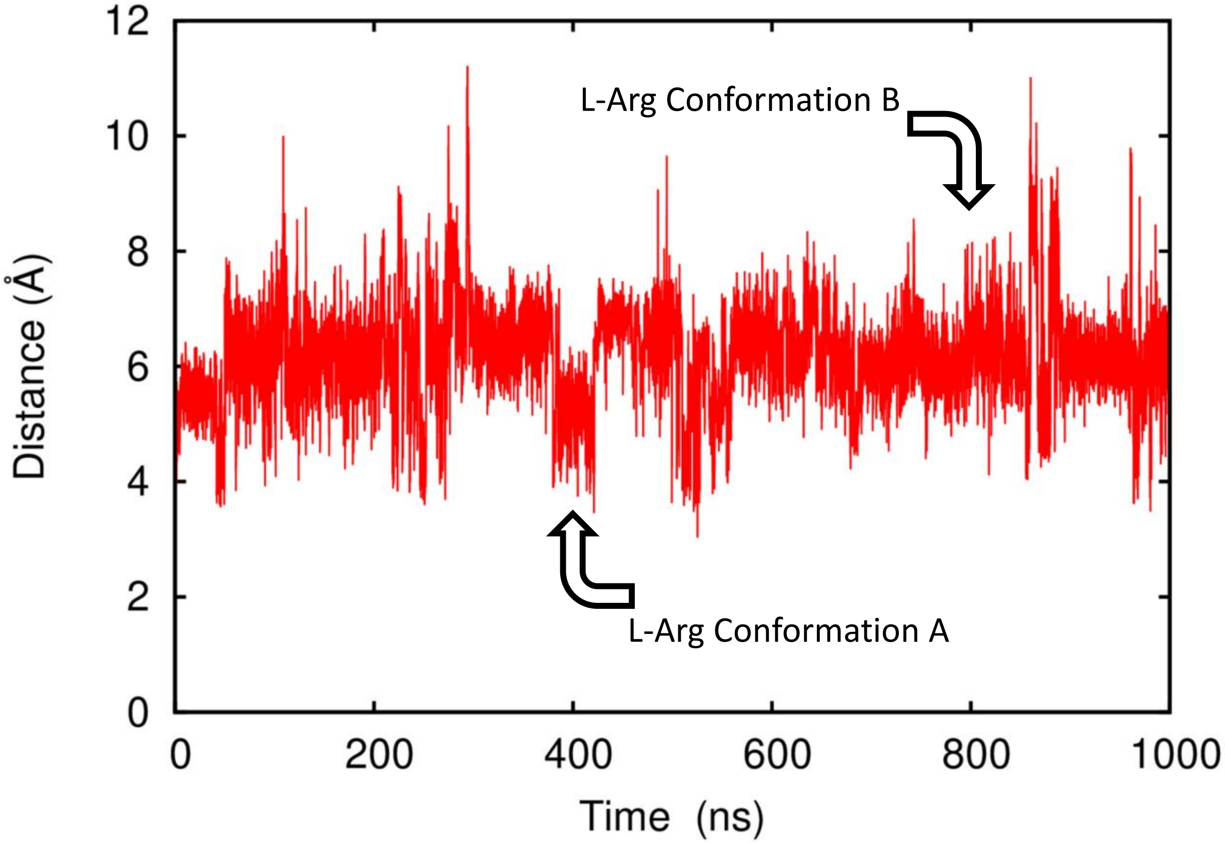

Figure S8. Conformations of L-Arg in R171A EFE. Distance between Fe and the C5-hydrogen of the L-Arg indicates the existence of both conformation A and B from the MD simulation of the R171A variant of EFE.

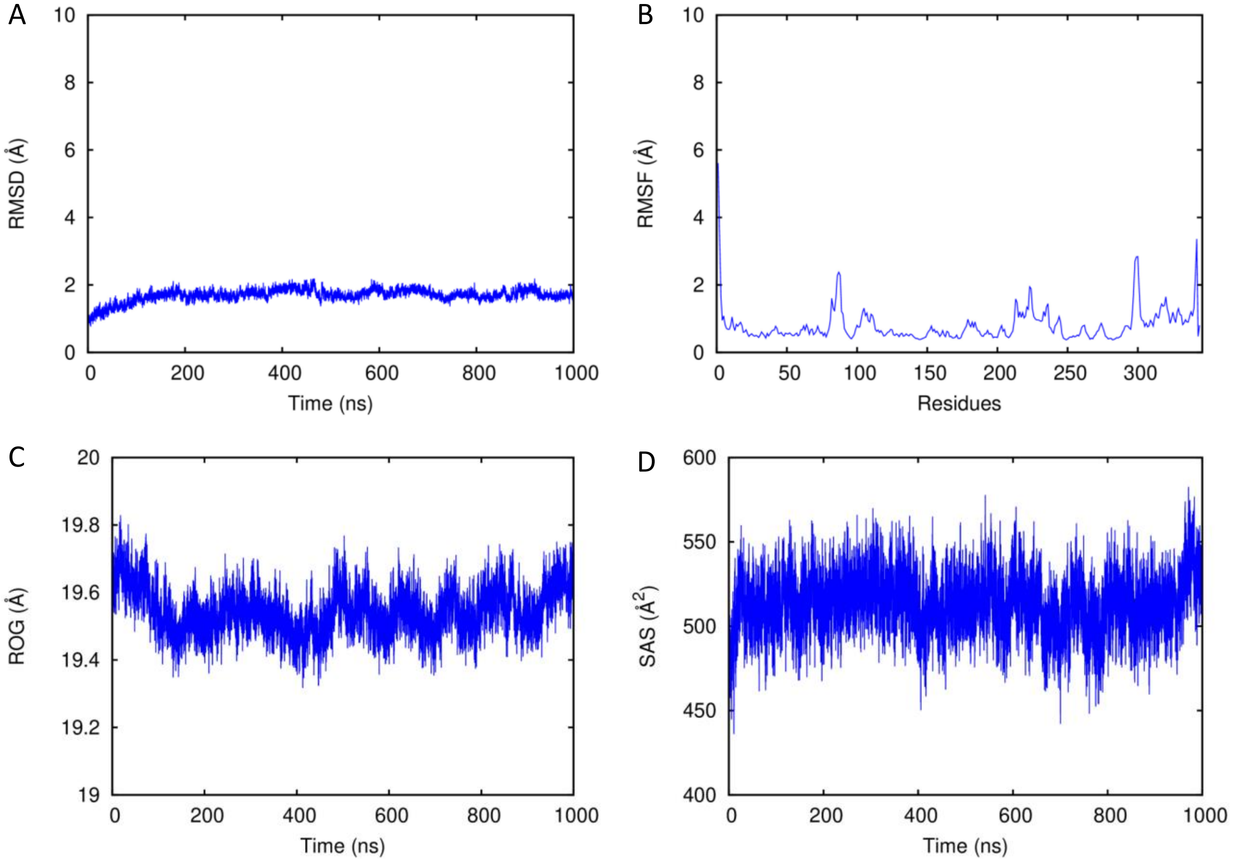

Figure S9. MD analysis of the Y306A variant of EFE in the absence of L-Arg, generated by using PDB: 6CF3. A) RMSD of the α carbons suggests the system is equilibrated. B) RMSF indicates the flexible regions of the protein. C) ROG shows stability of the overall protein fold. D) SAA showing evidence of an equilibrated system.

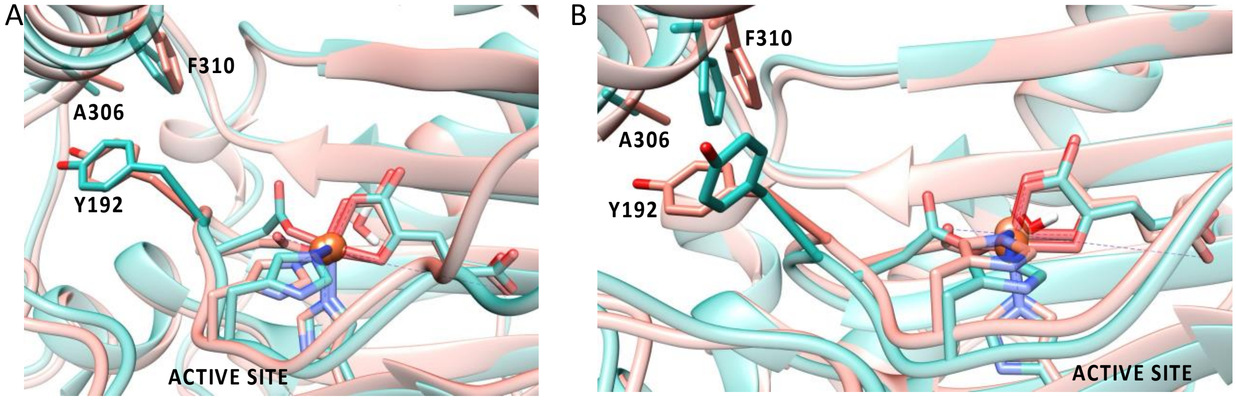

Figure S10. Different orientations of Y192 and F310 in Y306A EFE·Fe(II)·2OG as simulated by MD. Overlayed snapshots from the MD simulation showing A) Similar orientation of Y192 and F310 with those in the crystal structure of Y306A. B) Different orientation of Y192 and F310 compared to the crystal structure. Mn(II) in the crystal structure of Y306A was replaced with Fe(II). The crystal structure is represented in red, and the MD snapshots are shown in green.

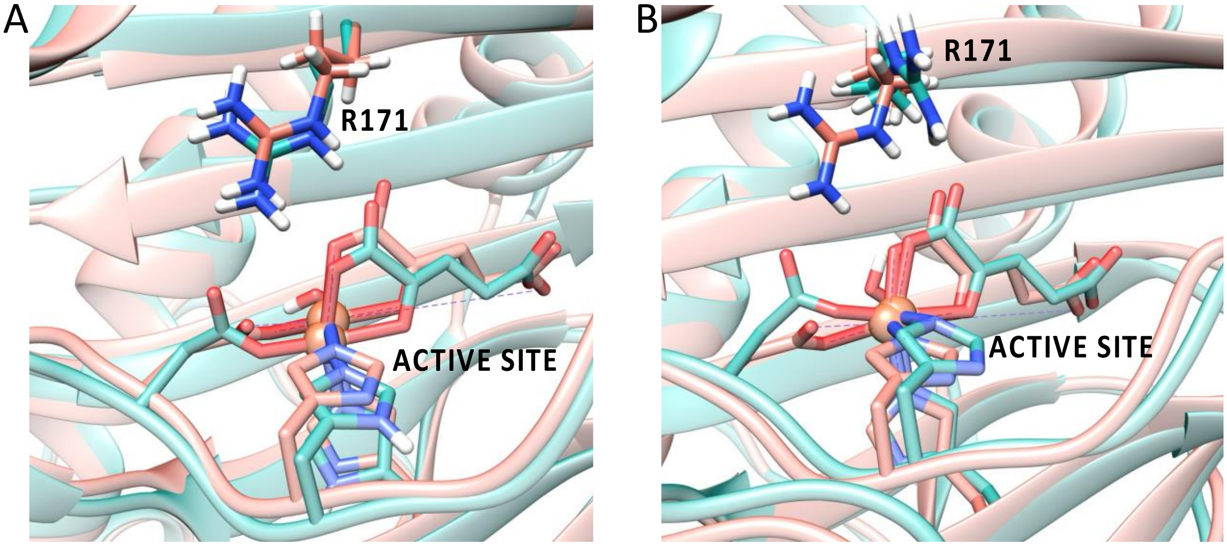

Figure S11: Different orientations of the side chain R171 in the dynamics of Y306A EFE·Fe(II)·2OG. Overlayed snapshots from the MD simulation showing A) Similar orientation of R171 with that in the crystal structure of Y306A. B) Different orientation of R171 with the crystal structure of Y306A. Mn(II) in the crystal structure was replaced with Fe(II). The crystal structure is represented in red, and the MD snapshots are shown in green.

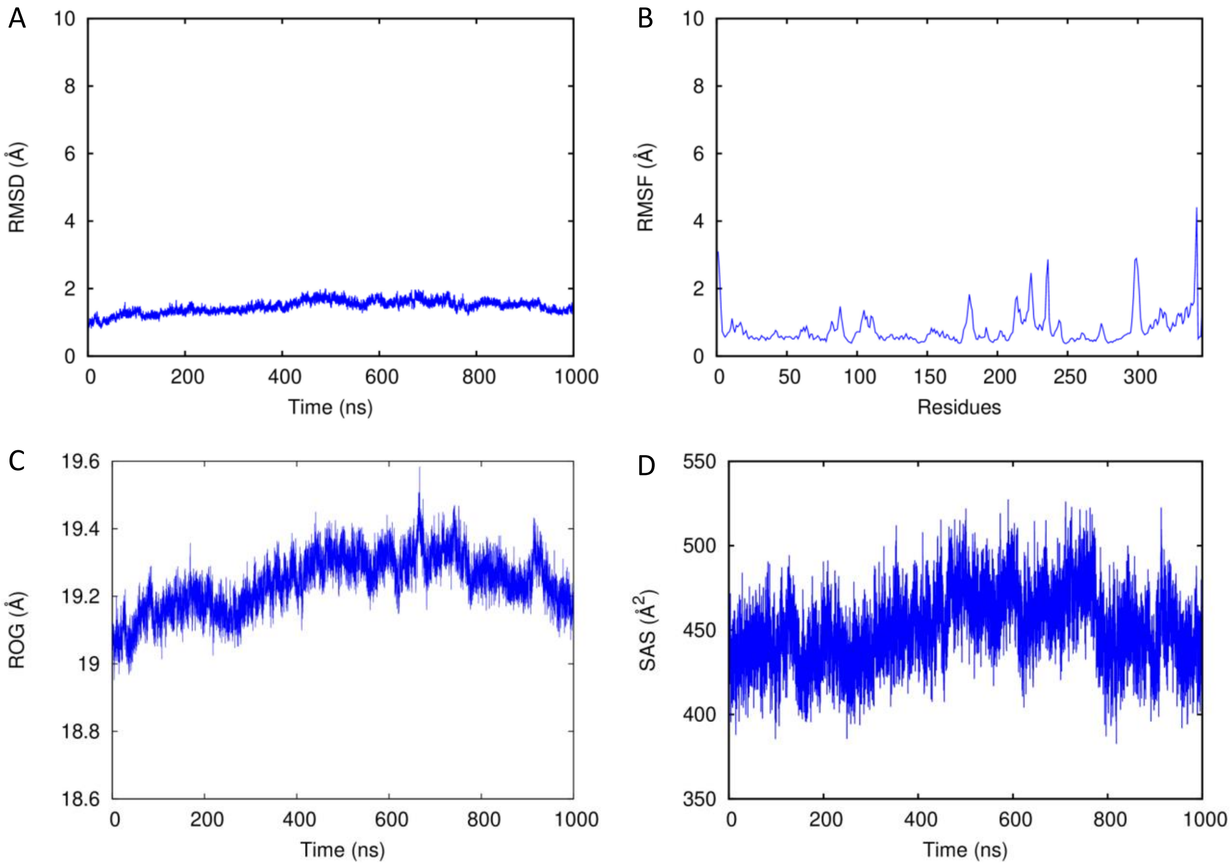

Figure S12. MD analysis of the Y306A EFE·Fe(III)·OO•$^{-}$·2OG·L-Arg, generated from the WT EFE structure with bound 2OG and L-Arg (PDB: 5V2Y). A) RMSD of the α carbons suggests the system is equilibrated. B) RMSF indicates the flexible regions of the protein. C) ROG shows stability of the overall protein fold. D) SAA showing evidence of an equilibrated system.

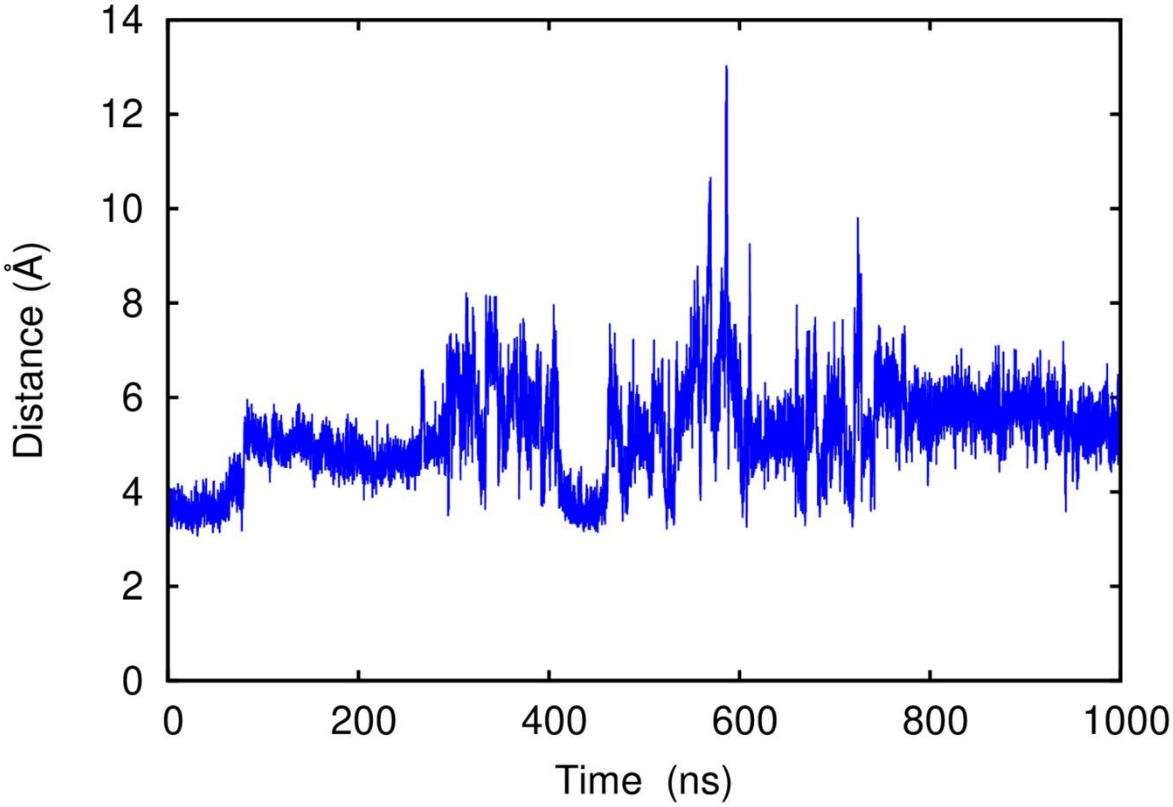

Figure S13. The distance between the CZ atom of L-Arg and R171 demonstrates weaker electrostatic interactions.

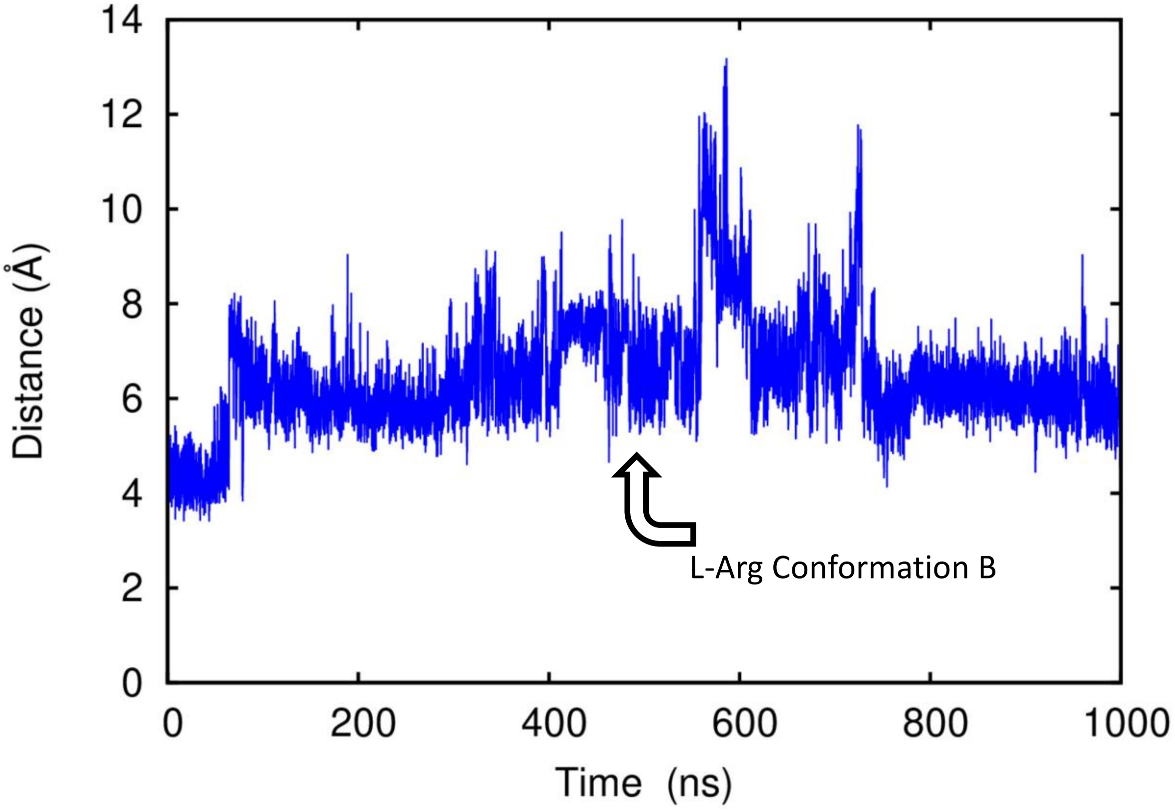

Figure S14. Conformations of L-Arg in Y306A EFE. Distance between Fe and the C5-hydrogen of L-Arg indicates a preference for conformation B from the MD simulation of the Y306A variant of EFE.

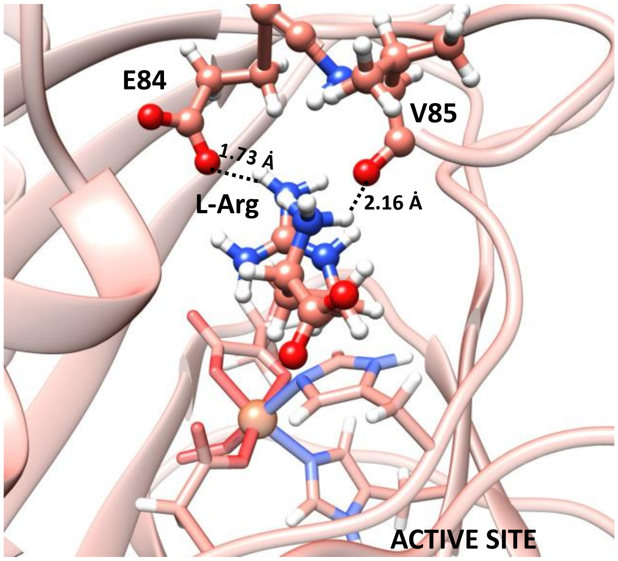

Figure S15. Representation of the hydrogen bonding interactions that stabilize L-Arg binding in the Y306A variant of EFE.
